# Inter-species and individualized biotransformation of five saponins by human being- and mouse-derived fecal microbiota

**DOI:** 10.1186/s13020-025-01190-2

**Published:** 2025-08-25

**Authors:** Wenjing Wei, Mingxiao Li, Lingyun Pan, Mijia Shao, Xiaofang He, Yuanyuan Li, Lili Sheng, Ningning Zheng, Houkai Li

**Affiliations:** 1 School of Pharmacy, Shanghai University of Traditional Chinese Medicine, Shanghai, 201203 China; 2 Institute of Pediatric Infection, Immunity, and Critical Care Medicine, Shanghai Children’s Hospital, Shanghai Jiao Tong University School of Medicine, Shanghai, 200062 China; 3 Experiment Center for Science and Technology, Shanghai University of Traditional Chinese Medicine, Shanghai, 201203 China

**Keywords:** Saponins, Gut microbiota, Biotransformation, Species-specific differences, Inter-individual variability

## Abstract

**Background:**

The gut microbiota plays a critical role in the biotransformation of saponins. However, current research predominantly focuses on metabolism by single microbial species, with limited investigation into inter-species differences or inter-individual variability in saponin biotransformation, especially by the culture of mixed gut microbiota. This study aims to elucidate the species-specific differences and inter-individual variability in gut microbiota-mediated saponin biotransformation through multidimensional analysis.

**Methods:**

In this study, we selected five representative saponins, including ginsenoside Rb1, ginsenoside Re, glycyrrhizic acid, saikosaponin D and dioscin, and conducted anaerobic cultures ex vivo with mixed gut microbiota derived from mice and humans. Metabolic profiles of parent compounds and their metabolites were analyzed using UPLC-MS/MS. Additionally, three saponins (ginsenoside Rb1, glycyrrhizic acid and saikosaponin D) were co-cultured with gut microbiota from 50 healthy volunteers to assess inter-individual biotransformation variability. 16S rRNA gene sequencing was employed to identify key microbial taxa and potential metabolic pathways.

**Results:**

We revealed distinct biotransformation patterns of multi-component saponins by mixed gut microbiota, with notable inter-individual variability observed among 50 healthy volunteers. Particularly, ginsenoside Rb1 exhibited the most significant individual differences in gut microbiota biotransformation. Further analysis demonstrated that the biotransformation capacity of gut microbiota was closely correlated with both its taxonomic composition and the relative abundances of specific bacterial genera.

**Conclusion:**

This study elucidated the pivotal role of gut microbiota in mediating inter-individual differences in saponin biotransformation, while having identified potential microbial communities and metabolic pathways involved in saponin biotransformation. These findings not only advance understanding of species-dependent biotransformation of saponins but also establish a foundation for screening microbial strains or enzymes to optimize saponin-derived therapies, thereby facilitating precision medicine and translational research.

**Graphical Abstract:**

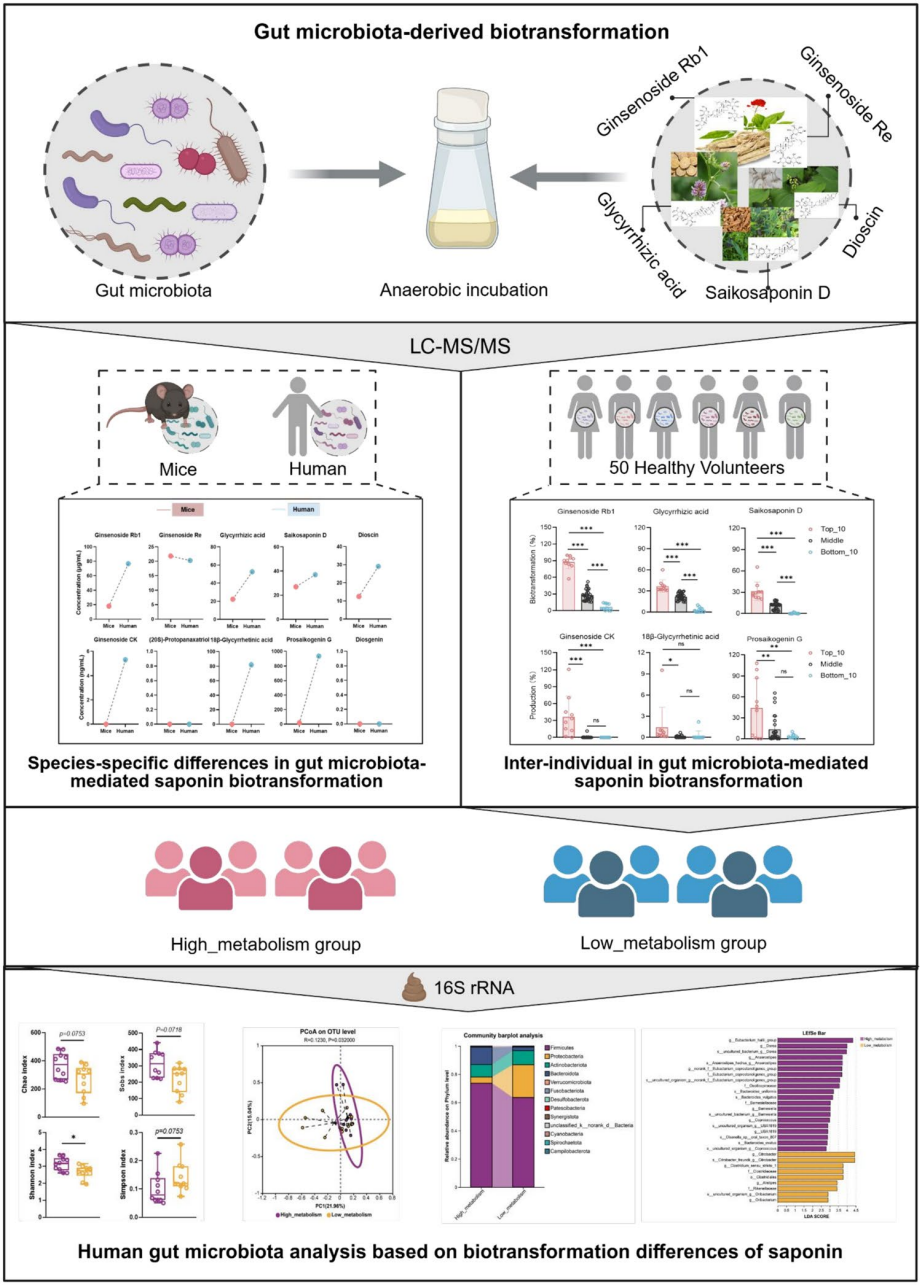

**Supplementary Information:**

The online version contains supplementary material available at 10.1186/s13020-025-01190-2.

## Introduction

The gut microbiota, often referred to as the “second genome” of the human body, encodes 150-fold more genes than the human genome and plays an indispensable role in drug metabolism [1–3]. Studies have demonstrated that gut microbiota directly participates in the biotransformation of drugs through enzymatic reactions (e.g., hydrolysis, reduction, dehydroxylation), significantly modulating their bioavailability and pharmacological activity [4, 5]. A representative example is the specific modification of saponins structures in natural products mediated by microbial-derived β-glucosidase. This enzyme system facilitates the conversion of poorly absorbed saponins such as ginsenoside Rb1 into highly bioactive sapogenin forms [6]. This bioconversion process has been established as a critical step in the clinical efficacy manifestation of various phytopharmaceutical active constituents [7–9]. Notably, the metabolic capacity of the gut microbiota is not only species-specific but also highly heterogeneous due to individual differences in microbiota composition, providing an important perspective for explaining individual differences in drug efficacy and toxicity [10–14].

Natural products (e.g., saponins) have diverse pharmacological activities and hold a significant position in drug development [15, 16], but their clinical application is often limited by low solubility and inter-individual metabolic differences [17]. The gut microbiota, as a crucial biotransformation medium, can optimize the pharmacological effects of these compounds through enzymatic reactions [3]. Current research predominantly focuses on the biotransformation of specific saponins by single bacterial species [18], particularly rodent-derived gut microbiota, while systematic comparisons of biotransformation differences between human- and mouse-origin gut microbiota remain scarce. Moreover, significant disparities exist between human and rodent gut microbiota in terms of species abundance and functional gene distribution [19]. Although studies have explored gut microbiota-mediated personalized biotransformation of saponins, most suffer from limited sample sizes and focus on the specific saponin [20–22], failing to comprehensively elucidate population-level metabolic heterogeneity or establish correlations between microbial composition and metabolic phenotypes [23, 24], thereby hindering their application in personalized medicine. Furthermore, mixed gut microbiota models bridge the gap between reductionist single-strain studies and real-world, individualized microbial communities, which is essential to unravel the ecological and functional complexity of saponin metabolism. However, the complexity of the gut microbiome and the lack of reliable mixed bacterial culture methods render the precise characterization of ex vivo saponin biotransformation by gut microbiota a challenging endeavor.

This study aims to elucidate species-specific differences and inter-individual variability in gut microbiota-mediated saponin biotransformation through multi-dimensional analyses. Utilizing an optimized novel culture medium (GB medium) developed from our previously established multi-dimensional evaluation system [25], which maximally simulates in situ host gut microbiome conditions, we selected five representative saponins (ginsenoside Rb1, ginsenoside Re, glycyrrhizic acid, saikosaponin D and dioscin) to systematically compare ex vivo biotransformation differences between human- and mouse-origin gut microbiota. By analyzing fecal microbiota from 50 healthy volunteers, we further investigated interindividual heterogeneity in saponin biotransformation phenotypes. Through integrated UPLC-MS/MS analysis and 16S rRNA sequencing, we identified species-specific differences and inter-individual variability in saponin biotransformation, proposing candidate microbial strains and metabolic pathways. These findings not only advance understanding of species-dependent biotransformation of saponins but also establish a foundation for screening microbial strains or enzymes to optimize saponin-derived therapies, thereby facilitating precision medicine and translational research.

## Methods

### Collection and processing of fecal samples from healthy volunteers and mice

Fecal samples were collected from 50 healthy volunteers (25 males and 25 females, aged 18–31 years, Table S1). All participants confirmed the absence of gastrointestinal diseases and had not taken antibiotics for at least two months prior to sample collection. The study protocol was approved by the Ethics Committee of Shanghai Hudong Hospital, China (Approval Number: 2022SHHDKY0602). Written informed consent was obtained from all participants prior to their involvement in the study. Additionally, fecal samples were collected from five healthy adult C57BL/6 J mice (weighing 18–22 g). Fresh fecal samples were suspended in PBSc solution (sterile phosphate-buffered saline containing 0.1% L-cysteine) at a ratio of 1:15 (feces: PBSc) under anaerobic conditions. The suspension was allowed to settle for 5 min to separate insoluble particles, after which the supernatant was collected for immediate culture [12]. The remaining fecal samples were stored at − 80 °C for 16S rRNA gene sequencing.

### Gut microbiota-mediated saponin biotransformation assay from 5 mice and 10 healthy volunteers ex vivo

Glycerol stock solutions from 5 mice and 10 healthy volunteers were selected and added to GB [25] at a ratio of 1:100 in an anaerobic chamber (Table S2, Table S3), and incubated in an anaerobic chamber at 37 °C for 24 h. Mixed cultures of human and mouse gut microbiota were obtained, respectively. Subsequently, the two sets of mixed cultures were combined with five saponins (ginsenoside Rb1, ginsenoside Re, glycyrrhizic acid, saikosaponin D and dioscin) at the same 1:100 ratio (each drug at a concentration of 200 μM in GB). In addition, each compound was also incubated in a microbiome-free GB control. Both the experimental and control groups were incubated under the same conditions for 24 h. After incubation, cultures were centrifuged (10,000 rpm, 1 min), and the supernatant was collected [12]. The supernatant samples (450 μL) were deproteinized with acetonitrile (450 μL), vortexed for 10 s, and centrifuged at 12,000 rpm for 20 min. The resulting supernatant was transferred to LC vial for the quantification of each drug prototype and metabolite.

### Growth experiment of mixed gut microbiota from humans and mice with saponin intervention ex vivo

Mixed cultures from two different species were combined with three saponins (ginsenoside Rb1, glycyrrhizic acid and saikosaponin D) at a ratio of 1:100 and added to GB (the concentration of each drug in GB was 200 μM). After inoculation, the bacterial suspension was inverted to ensure thorough mixing. Baseline samples were then collected from the culture tubes, followed by anaerobic incubation for 48 h. Bacterial suspension samples were collected at different time points to measure OD600. The supernatant for OD600 readings was obtained after centrifugation (12,000 rpm/min, 2 min). ΔOD600 was calculated as the difference between baseline and final readings.

### Gut microbiota-mediated saponin biotransformation assay from 50 healthy volunteers ex vivo

Under anaerobic conditions, glycerol stock solutions of fecal samples from 50 healthy volunteers were added to GB medium at a 1:100 ratio, respectively, and incubated at 37 °C for 24 h in an anaerobic chamber. Subsequently, the cultures and the three saponins (ginsenoside Rb1, glycyrrhizic acid and saikosaponin D) were added to the GB in the same 1:100 ratio (200 μM of each drug in GB). Additionally, each drug was incubated in a microbiome-free GB control under the same conditions. Both experimental and control samples were incubated for 24 h under the same conditions. After incubation, cultures were centrifuged (10,000 rpm,1 min), and the supernatant was collected. The supernatant samples (450 μL) were deproteinized with acetonitrile (450 μL), vortexed for 10 s, and centrifuged at 12,000 rpm for 20 min. The resulting supernatant was transferred to LC vial for the quantification of each drug prototype and metabolite.

### UPLC-MS/MS analysis

The UPLC-MS/MS system included an Acquity ultra-high-performance liquid chromatography (UPLC) system (Waters, USA) coupled with an API 6500 QTRAP triple quadrupole-linear ion trap mass spectrometer equipped with an electrospray ionization (ESI) source (AB SCIEX, USA). Chromatographic separation was achieved using an ACQUITY UPLC^®^ BEH C18 column (100 mm × 2.1 mm, 1.7 μm particle size) maintained at 35 °C, with a sample injection volume of 0.4 μL. The mobile phase consisted of 2 mM ammonium acetate in water with 0.1% formic acid (solvent A) and acetonitrile (solvent B). Gradient elution was performed at a flow rate of 0.3 mL/min under the following conditions: Chromatographic Condition: 0–0.5 min, 84% A; 0.5–1 min, 70% A; 1–7.5 min, 25% A; 7.5–9.5 min, 5% A; 9.5–11.5 min, 84% A. Following chromatographic separation, the eluent was introduced into the mass spectrometer via an electrospray ionization (ESI) ion source. The ion source parameters were set as follows: ion source temperature (TEM) at 550 °C, nebulizer gas (Ion Source Gas 1, GS1) at 55.0 psi, auxiliary heating gas (Ion Source Gas 2, GS2) at 55.0 psi, curtain gas (CUR) at 35.0 psi, and ion spray voltage (IS) at -4500.0 V (Negative Mode) or 5500.0 V (Positive Mode). The detailed mass spectrometry parameters are provided in Table S4.

### 16S rRNA gene sequencing

Sequencing was performed by Shanghai Majorbio Bio-pharm Technology Co., Ltd. (Shanghai, China). Total DNA was extracted from fecal samples of 50 healthy volunteers using the E.Z.N.A.^®^ Soil DNA Kit (Omega Bio-tek, Norcross, GA, USA). The bacterial 16S rRNA gene fragments (V3-V4) were amplified from the extracted DNA using primers 338F (5′-ACTCCTACGGGAGGCAGCAG-3′) and 806R (5′-GGACTACHVGGGTWTCTAAT-3′), with PCR conditions (30 s at 95 °C, 30 s at 55 °C, and 45 s at 72 °C, for a total of 27 cycles). The amplified fragments were sequenced using the Illumina MiSeq PE300 system (Illumina, San Diego, USA) according to the standard protocol. The raw data were uploaded to the NCBI SRA database. Data analysis was performed using the free online platform of Majorbio Cloud Platform (https://cloud.majorbio.com). The Principal Coordinates Analysis (PCoA) based on Bray–Curtis dissimilarity was conducted to visualize the community similarity and overall differences in gut microbiota between two groups; the statistical significance of group differences was assessed using the Analysis of Similarities (ANOSIM) test. For functional prediction analysis, we used PICRUSt2 (version: 2.2.0), which relies on the KEGG (Kyoto Encyclopedia of Genes and Genomes) database as its reference to predict the abundances of KEGG Ortholog (KO) abundances and map them to KEGG pathways, based on our processed 16S rRNA gene amplicon sequencing data (ASVs/OTUs and abundance table).

### Statistical analysis

Data are presented as mean ± SEM. For multiple-group comparisons, one-way ANOVA followed by Tukey’s post hoc test was used. Differences between two groups were compared using the Mann–Whitney U test. *P* < 0.05 was considered statistically significant. Analyses were performed using GraphPad Prism 8.0 (GraphPad, La Jolla, CA, USA).

## Results

### UPLC-MS/MS detection of five saponins

**Fig. 1:**
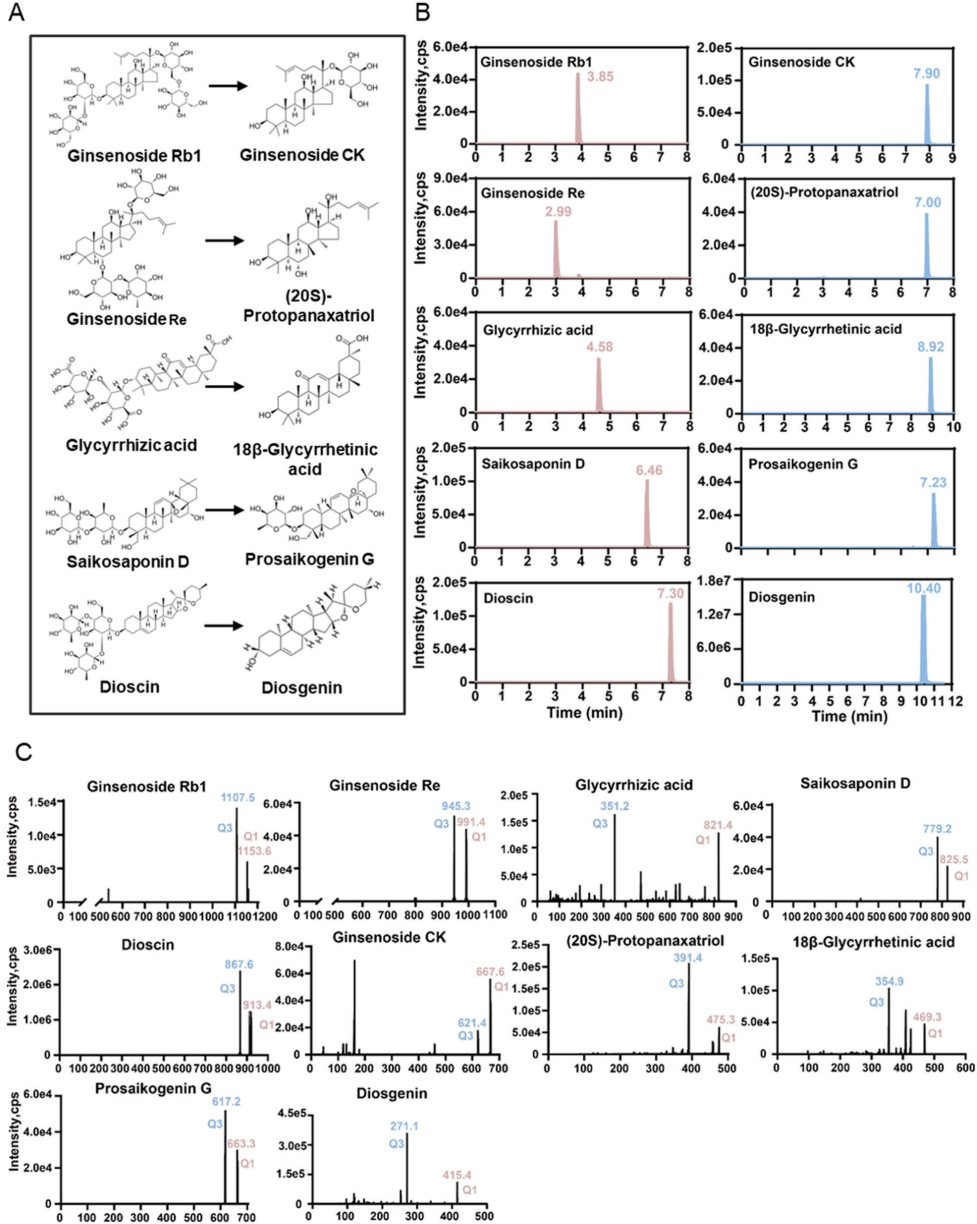
UPLC-MS/MS method of five saponin components and their metabolites. **A** Metabolism of Ginsenoside Rb1, Ginsenoside Re, Glycyrrhizic acid, Saikosaponin D and Dioscin. **B** The chromatograms of Ginsenoside Rb1, Ginsenoside CK, Ginsenoside Re, (20S)-Protopanaxatriol, Glycyrrhizic acid, 18β-Glycyrrhetinic acid, Saikosaponin D, Prosaikogenin G, Dioscin and Diosgenin. **C** Ion pairs of Ginsenoside Rb1, Ginsenoside CK, Ginsenoside Re, (20S)-Protopanaxatriol, Glycyrrhizic acid, 18β-Glycyrrhetinic acid, Saikosaponin D, Prosaikogenin G, Dioscin and Diosgenin

In this study, we firstly established a UPLC-MS/MS method for the detection of ginsenoside Rb1, ginsenoside Re, glycyrrhizic acid, saikosaponin D and dioscin in the supernatant of culture medium samples. The biotransformation of the five saponins is illustrated in Fig. 1A, while the chromatograms and ion pairs of the five saponins and their metabolites are shown in Fig. 1B and C, respectively.

### Species-specific differences in gut microbiota-mediated saponin biotransformation

**Fig. 2:**
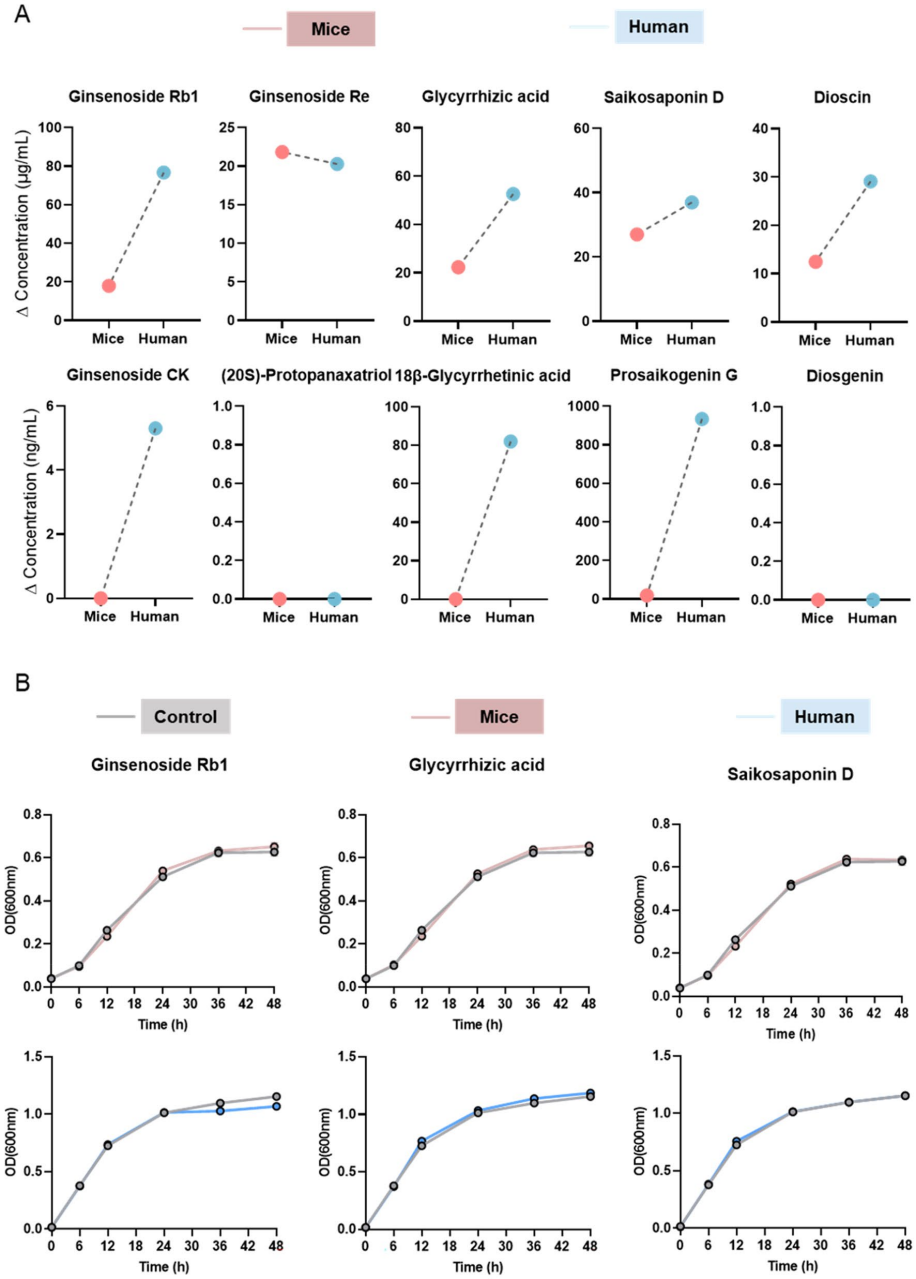
Interactions between different species of gut microbiota and saponin components ex vivo. **A** Drug concentration after incubation with gut microbiota on 24 h for each species-drug combination, with the y-axis representing the change in Ginsenoside Rb1, Ginsenoside Re, Glycyrrhizic acid, Saikosaponin D and Dioscin concentration (ΔC, C_0 h_–C_24 h_) and their metabolites (Ginsenoside CK, (20S)-Protopanaxatriol, 18β-Glycyrrhetinic acid, Prosaikogenin G and Diosgenin) concentration (ΔC, C_24 h_–C_0 h_). **B** Growth curves after incubation with gut microbiota on 0, 6, 12, 24, 36 and 48 h for each species-drug combination. Data are presented as mean ± SEM. (*n* = 3)

Based on the “host in situ gut microbiota culture” technique previously established by our team [25], we first explored the effects of mixed bacteria from different species on the biotransformation of five saponins ex vivo, including ginsenoside Rb1, ginsenoside Re, glycyrrhizic acid, saikosaponin D and dioscin. Figure 2A shows that after 24 h of co-incubation with mixed human and mouse gut microbiota, the ∆C values (C_24 h_–C_0 h_) of five saponins showed a change, indicating that these saponins underwent biotransformation to varying degrees in the presence of both human and mouse gut microbiota. The contents of 20(S)-protopanaxatriol and diosgenin in the incubation solutions of human and mouse bacteria were below the detection limit, indicating that ginsenoside Re and dioscin may produce other metabolites after being metabolized by gut microbiota. Furthermore, the contents of ginsenoside CK, 18β-glycyrrhetinic acid and saikosaponin G in the mouse bacterial incubation solution were below the detection limit, while these three metabolites were detected in the human bacterial incubation solution at 24 h, suggesting that human gut microbiota is involved in the biotransformation of ginsenoside Rb1, glycyrrhizic acid and saikosaponin D to ginsenoside CK, 18β-glycyrrhetinic acid and saikosaponin G.

Ginsenoside Rb1, glycyrrhizic acid and saikosaponin D, which exhibit metabolic differences between human and mouse gut microbiota, were further selected as the subjects of our study. We aimed to investigate whether these three saponins have any impact on the growth of mixed bacteria from humans and mice ex vivo. The growth curves (from 0 to 48 h) showed no differences between the control and bacteria-treated groups, indicating that the three saponins did not affect the growth of the mixed bacteria from humans and mice ex vivo **(**Fig. 2B**)**.

### Inter-individual in gut microbiota-mediated saponin biotransformation

**Fig. 3:**
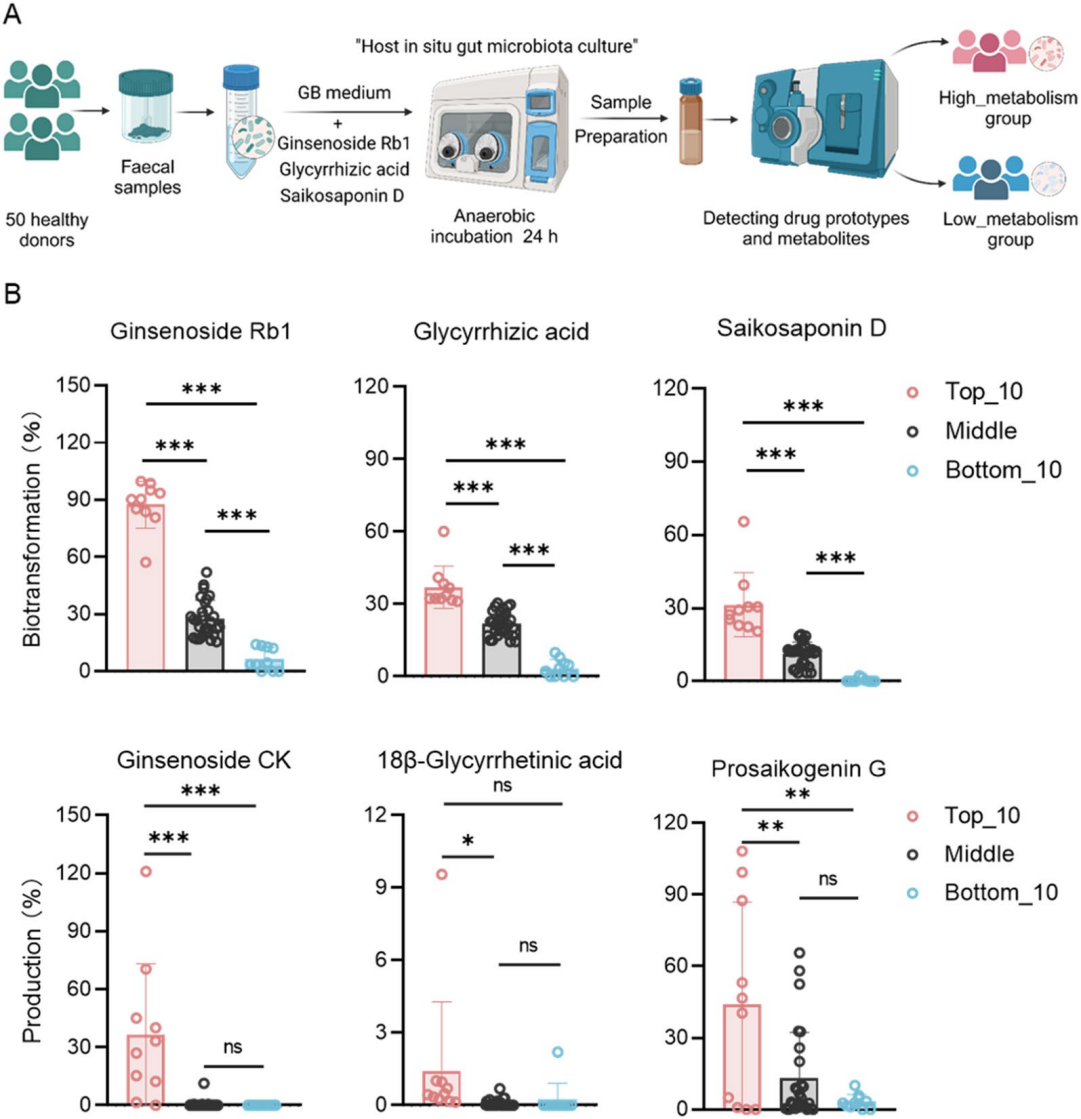
Personalized drug metabolism by human gut microbiota ex vivo. **A** Flow chart of the experiment. **B** The figure illustrates the biotransformation capacity of gut microbiota to metabolize the prototype drug into metabolites over a 24 h incubation period for each donor-drug combination. Prototype drug biotransformation (%) was quantified as (C_0 h_–C_24 h_)/C_0 h_ × 100%, where C_0 h_ and C_24 h_ represent the prototype drug concentrations at 0 h and 24 h, respectively. Metabolite formation (%) was calculated as (C_24 h_–C_0 h_)/C_0 h_ × 100%, where C_0 h_ and C_24 h_ correspond to metabolite concentrations at 0 h and 24 h, respectively. Data are presented as mean ± SEM. Statistical significance was assessed by one-way ANOVA, followed by Tukey’s post hoc test. ^*^P < 0.05, ^**^P < 0.01. ^***^P < 0.001

In order to investigate the inter-individual variability of gut microbiota-mediated biotransformation of saponins, we further employed our previously established ex vivo anaerobic cultivation model of gut microbiota to quantify the biotransformation patterns of three representative saponins: ginsenoside Rb1, glycyrrhizic acid and saikosaponin D in microbiota from 50 healthy human donors. The experimental design is illustrated in Fig. 3A. By calculating the percentage of the prototype drug biotransformation within 0–24 h [(C_0 h_–C_24 h_)/C_0 h_ × 100%], we found that the gut microbiota of 50 donors exhibited varying degrees of interindividual variability in their biotransformation capacity for three saponins (ginsenoside Rb1, glycyrrhizic acid and saikosaponin D). Correspondingly, the production of the major bioactive metabolites (ginsenoside CK, 18β-glycyrrhetinic acid and saikosaponin G) also exhibited significant interindividual differences (Fig. 3B). Based on the biotransformation percentage of the parent drugs, we divided the donors into three groups: Top_10 (10 individuals with high biotransformation capacity), Middle group and Bottom_10 (10 individuals with low biotransformation capacity). Analysis revealed significant differences in the intestinal microbiota’s biotransformation capacity (measured by biotransformation percentage) for the three prototypical saponins between the Top_10 and Bottom_10 groups (Fig. 3B). Additionally, the trends in the three bioactive metabolites were highly consistent with the biotransformation capacity gradient of the corresponding prototypical drugs. These results indicate significant phenotypic differentiation between ‘efficient converters’ (Top_10) and ‘inefficient converters’ (Bottom_10) in gut microbiota-mediated saponin biotransformation among healthy individuals.

Additionally, we assessed the effects of donor sex and age on gut microbiota-mediated biotransformation of the three saponins ex vivo. No significant associations were observed between sex (Fig. S1A) or age (Fig. S1B) and microbial biotransformation rates in the 50 donors.

### Human gut microbiota analysis based on biotransformation differences of ginsenoside Rb1

To further explore the relationship between interindividual differences in the biotransformation of three saponins and gut microbiota composition, we selected fecal samples of the aforementioned Top_10 (high_metabolism) and Bottom_10 (low_metabolism) healthy volunteers and co-cultured them with three saponin compounds. Subsequently, 16S rRNA gene sequencing was used to evaluate the composition and diversity of gut microbiota between the high and low biotransformation capacity groups.

**Fig. 4:**
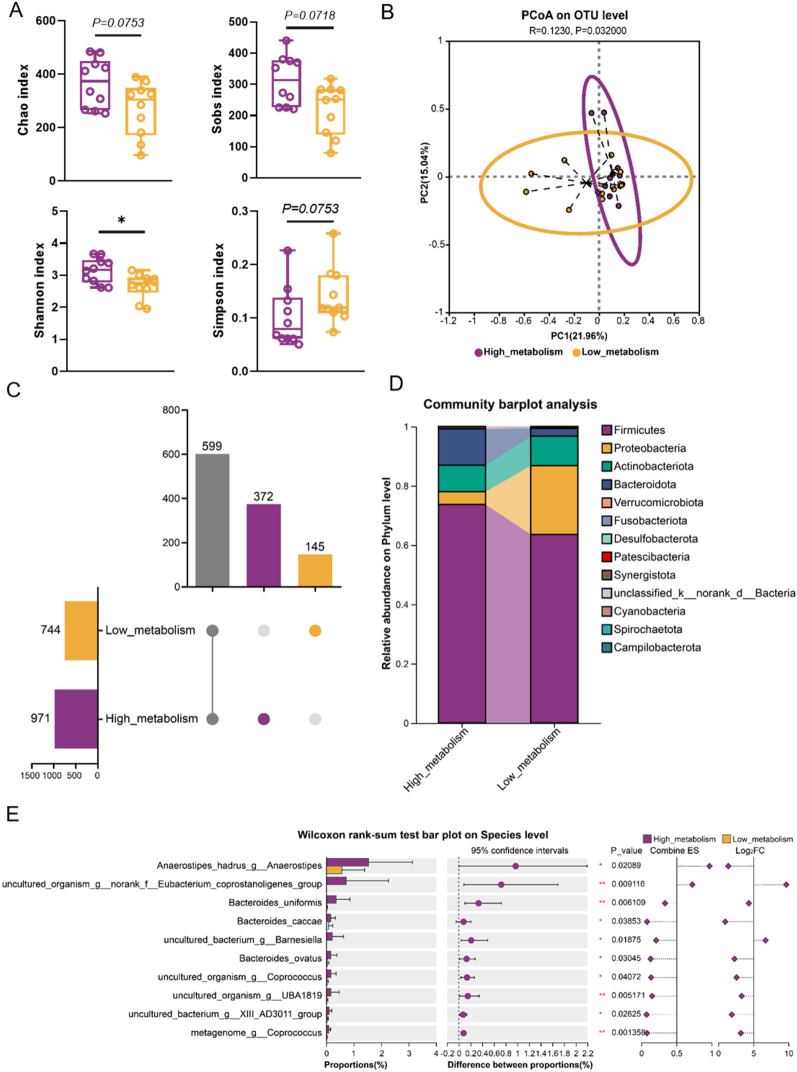
Analysis of human gut microbiota based on differences in biotransformation of Ginsenoside Rb1 ex vivo. **A** Alpha diversity analysis with the Mann–Whitney U test. **B** PCoA analysis at OTU level based on Bray–Curtis. **C** Upset diagram showing OTU overlap between high_metabolism group and low_metabolism group. **D** Gut microbiota change at phylum level (barplot). **E** Differentially abundant species (Wilcoxon rank-sum test). Exact P-values and 95% CIs are indicated in the figure. ^*^P < 0.05, ^**^P < 0.01; *n* = 10 per group

Analysis of human gut microbiota for differences in biotransformation of ginsenoside Rb1 ex vivo revealed that the Chao and Sobs indices were higher in the high_metabolism group compared to the low_metabolism group (Fig. 4A), indicating that individuals with high biotransformation capacity typically exhibit higher community richness. Additionally, the Shannon index was significantly higher in the high_metabolism group, while the Simpson index was lower than that in the low_metabolism group, suggesting that individuals with high biotransformation capacity tend to have higher microbial diversity (Fig. 4A). Beta diversity analysis using principal coordinates analysis (PCoA) revealed significant differences in the gut microbial community structure between the high_ and low_metabolism groups (Fig. 4B; R = 0.1230, *P* = 0.032000). Venn analysis showed that there were 372 unique microbial species in the high_metabolism group, 145 unique species in the low_metabolism group, and 599 shared species between the two groups (Fig. 4C). The effective sequences of all samples were annotated and classified, and the relative abundances of the top 20 taxa were analyzed. The results indicated that at the phylum level, the gut microbiota was predominantly composed of Firmicutes, Proteobacteria, Actinobacteriota and Bacteroidetes. Among these, the relative abundances of Firmicutes and Bacteroidetes were higher in the high_metabolism group compared to the low_metabolism group (Fig. 4D). At the family level, 9 families exhibited higher relative abundances in the high_metabolism group (Fig. S2A). At the genus level, 11 genera showed higher relative abundances in the high_metabolism group (Fig. S2B). Linear discriminant analysis effect size (LEfSe) analysis indicated that *Eubacterium coprostanoligenes_group, Oscillospiraceae* and *Barnesiellaceae* were representative differential microbes in the high_metabolism group (Fig. S2C). Intergroup difference analysis revealed that at the genus level, *Eubacterium_halli_group*, *Anaerostipes*, *norank_f_Eubacterium_coprostanoligenes_group*, *Coprococcus*, *Barnesiella*, *norank_f_Oscillospiraceae*, *UBA1819* and *Family_XIII_AD3011_group* had significantly higher relative abundances in the high_metabolism group (Fig. S2D, *P* < 0.05). At the species level, the relative abundances of *Anaerostipes_hadrus_g_Anaerostipes*, *uncultured_organism_g_norank_f_Eubacterium_coprostanoligenes_group*, *Bacteroides_uniformis*, *Bacteroides_caccae*, *uncultured_bacterium_g_Barnesiella*, *Bacteroides_ovatus*, *uncultured_organism_g_Coprococcus*, *uncultured_organism_9_UBA1819*, *uncultured_bacterium_9_XIII_AD3011_group* and *metagenome_g_Coprococcus* were significantly increased in the high_metabolism group (Fig. 4E, *P* < 0.05). Further correlation analysis revealed that the biotransformation ability of ginsenoside Rb1 was significantly positively associated with the relative abundance of the specific microbial taxa, including the genus *Barnesiella*, *Coprococcus*, *Family_XIII_AD3011_group*, *UBA1819*, *Eubacterium_halli_group*, and *Anaerostipes*, as well as the species *metagenome_g_Coprococcus, Anaerostipes_hadrus_g_Anaerostipes* and *Bacteroides uniformis* (Fig. S3, *P* < 0.05).

**Fig. 5:**
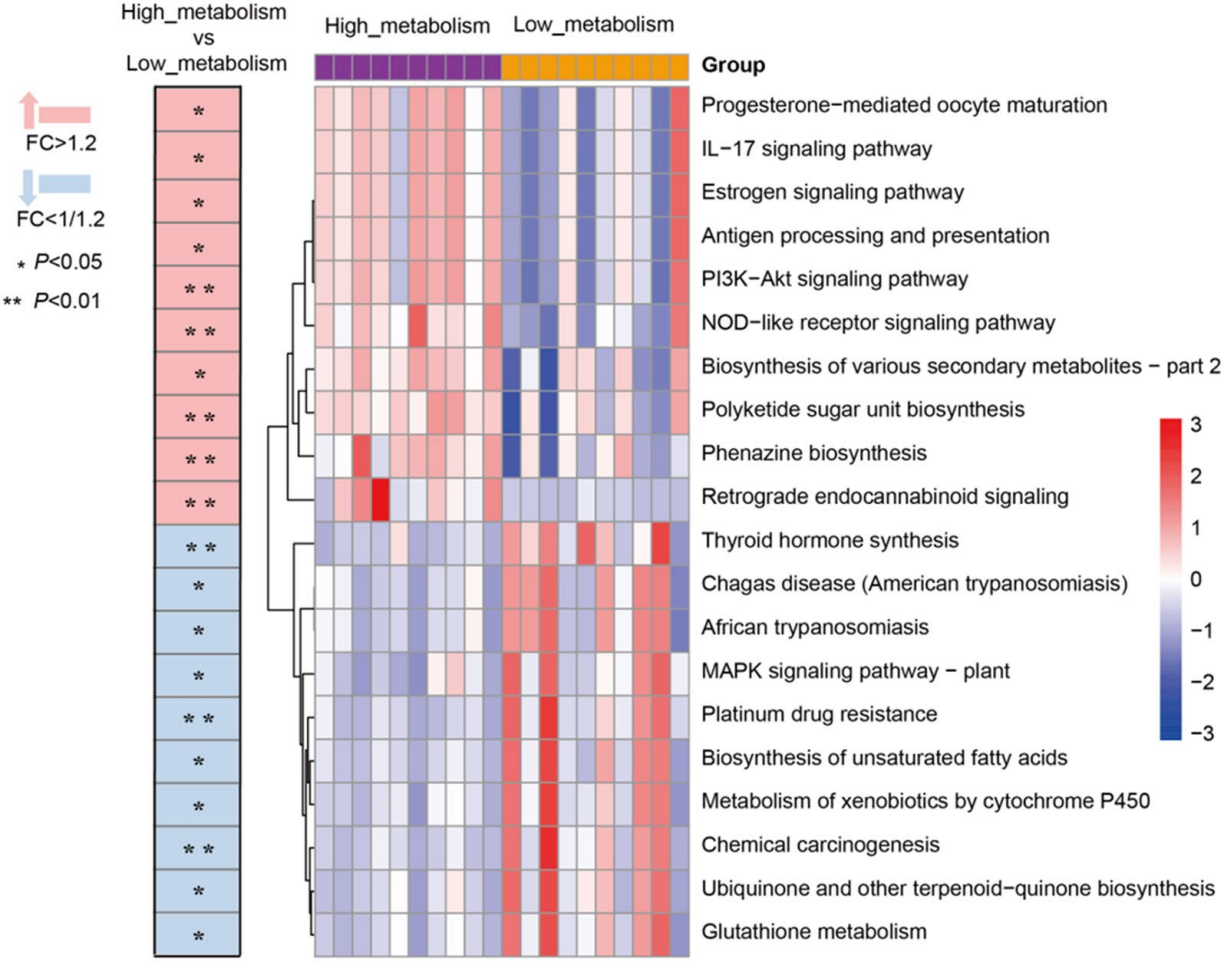
Heatmap summarizing the changes in gut microbial community function based on differences in biotransformation of Ginsenoside Rb1 ex vivo. (Pink color represents enrichment in high_metabolism group, blue color represents enrichment in low_metabolism group; ^*^P < 0.05, ^**^P < 0.01; *n* = 10 per group.)

Subsequently, we conducted predictive analysis of the enriched metabolic pathways in the gut microbiota and found significant differences in certain metabolic pathways between the two groups. Compared to the low_metabolism group, the high_metabolism group exhibited higher relative abundances in pathways such as progesterone-mediated oocyte maturation, IL-17 signaling pathway, estrogen signaling pathway, antigen processing and presentation, PI3K-Akt signaling pathway, NOD-like receptor signaling pathway, biosynthesis of various secondary metabolites—part 2, polyketide sugar unit biosynthesis, phenazine biosynthesis and retrograde endocannabinoid signaling (Fig. 5, *P* < 0.05). These results suggest that the differences in gut microbiota composition and diversity between the two groups could be related to their microbial metabolic functions.

### Human gut microbiota analysis based on biotransformation differences of glycyrrhizic acid

**Fig. 6:**
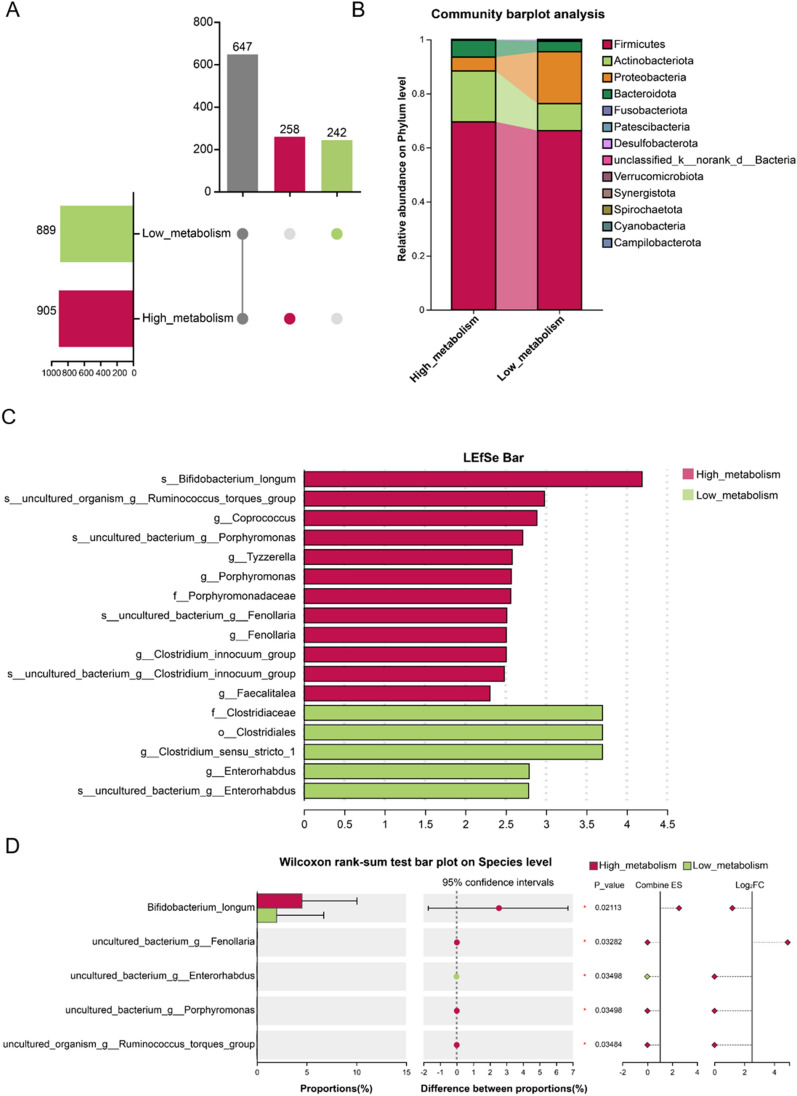
Analysis of human gut microbiota based on differences in biotransformation of Glycyrrhizic acid ex vivo. **A** Upset diagram showing OTU overlap between high_metabolism group and low_metabolism group. **B** Gut microbiota change at phylum level (barplot). **C** Gut microbiota structure difference LEfSe analysis. **D** Differentially abundant species (Wilcoxon rank-sum test). Exact P-values and 95% CIs are indicated in the figure. ^*^P < 0.05; *n* = 10 per group

Venn analysis revealed 258 unique microbial species in the high_metabolism group and 242 unique species in the low_metabolism group, with 647 species shared between the two groups (Fig. 6A). Analysis of species composition showed that, at the phylum level, the relative abundance of Proteobacteria was significantly higher in the high_metabolism group compared to the low_metabolism group (Fig. 6B). At the family level, nine families exhibited higher relative abundance in the high_metabolism group (Fig. S4A). At the genus level, 11 genera showed higher relative abundance in the high_metabolism group (Fig. S4B). LEfSe analysis indicated that *Porphyromonadaceae* was a representative differential microorganism in the high_metabolism group (Fig. 6C). Intergroup difference analysis revealed that, at the genus level, the relative abundances of *Tyzzerella*, *Fenollaria*, *Faecalitalea* and *Porphyromonas* were significantly higher in the high_metabolism group compared to the low_metabolism group (Fig. S4C, *P* < 0.05). At the species level, the relative abundances of *Bifidobacterium_longum*, *uncultured_bacterium_g_Fenollaria*, *uncultured_bacterium_g_Porphyromonas* and *uncultured_organism_g_Ruminococcus_torques_group* were significantly increased in the high_metabolism group (Fig. 6D, *P* < 0.05). Further correlation analysis indicated that the capacity for Glycyrrhizic acid biotransformation was strongly correlated with the relative abundance of the genus *Tyzzerella* and *Fenollaria* (Fig. S5, *P* < 0.05).

**Fig. 7:**
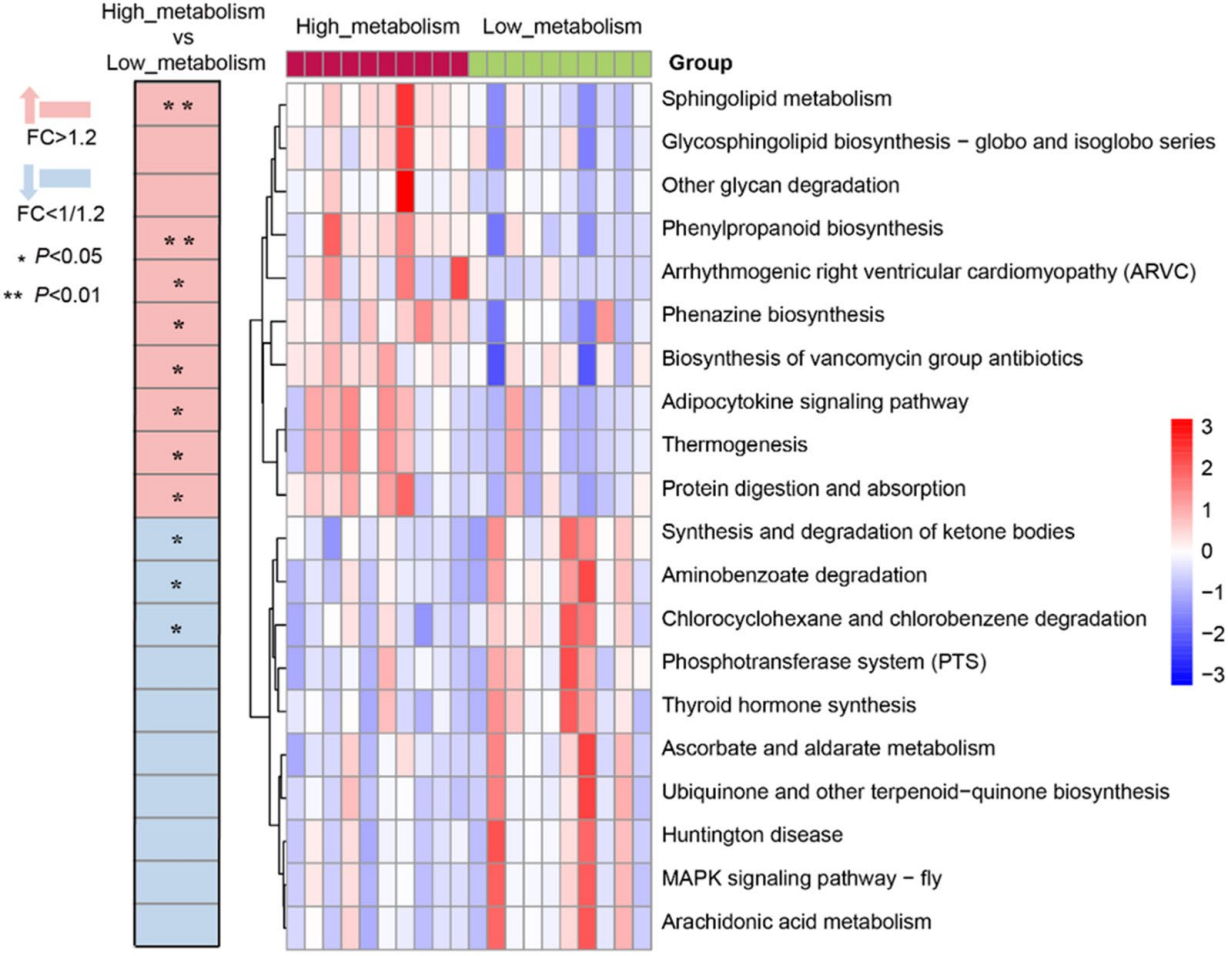
Heatmap summarizing the changes in gut microbial community function based on differences in biotransformation of Glycyrrhizic acid ex vivo. (Pink color represents enrichment in high_metabolism group, blue color represents enrichment in low_metabolism group; ^*^P < 0.05, ^**^P < 0.01; *n* = 10 per group.)

KEGG pathway analysis demonstrated that the relative abundances of metabolic pathways such as sphingolipid metabolism, phenylpropanoid biosynthesis, arrhythmogenic right ventricular cardiomyopathy (ARVC), phenazine biosynthesis, biosynthesis of vancomycin group antibiotics, adipocytokine signaling pathway, thermogenesis and protein digestion and absorption were higher in the high metabolism group compared to the low metabolism group (Fig. 7, *P* < 0.05).

### Human gut microbiota analysis based on biotransformation differences of saikosaponin D

**Fig. 8:**
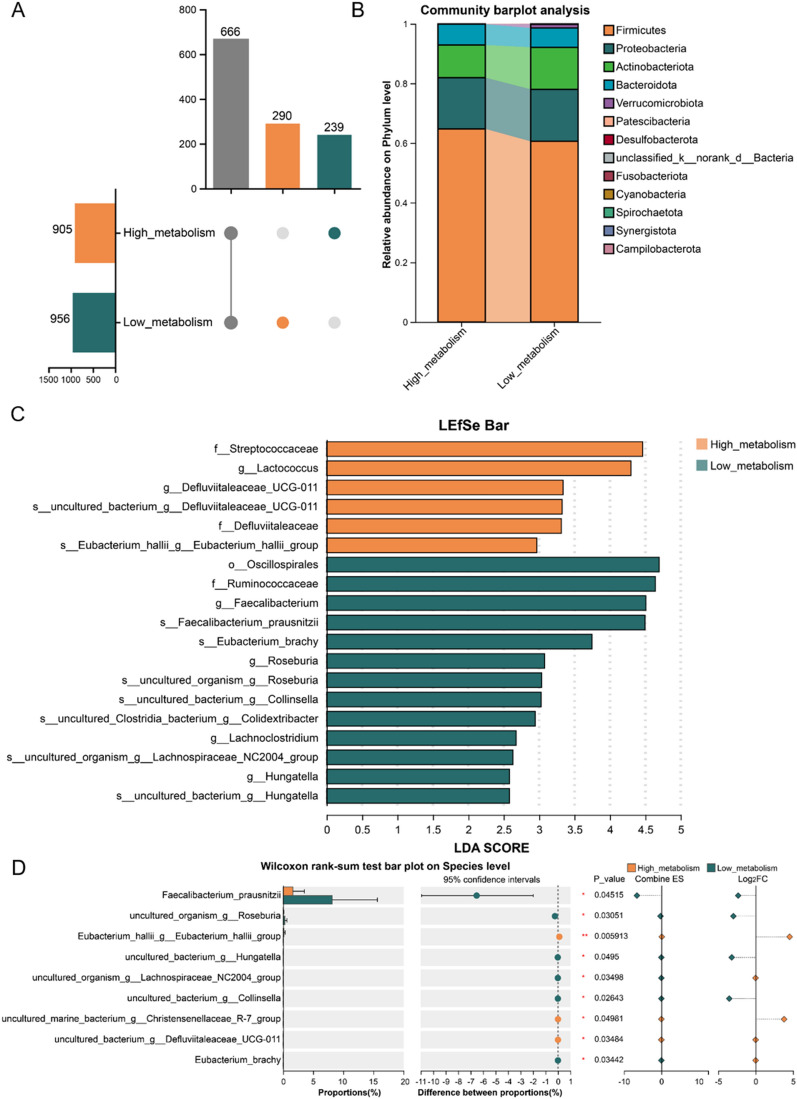
Analysis of human gut microbiota based on differences in biotransformation of Saikosaponin D ex vivo. **A** Upset diagram showing OTU overlap between high_metabolism group and low_metabolism group. **B** Gut microbiota change at phylum level (barplot). **C** Gut microbiota structure difference LEfSe analysis. **D** Differentially abundant species (Wilcoxon rank-sum test). Exact P-values and 95% CIs are indicated in the figure; ^*^P < 0.05, ^**^P < 0.01; *n* = 10 per group

Venn analysis identified 290 unique microbial species in the high_metabolism group and 239 unique species in the low_metabolism group, with 666 shared species between the two groups (Fig. 8A). Species composition analysis showed that, at the phylum level, the relative abundance of Firmicutes was significantly higher in the high_metabolism group than in the low_metabolism group (Fig. 8B). At the family level, six families exhibited higher relative abundance in the high_metabolism group (Fig. S6A). At the genus level, 11 genera showed higher relative abundance in the high_metabolism group (Fig. S6B). LEfSe analysis indicated that *Streptococcaceae* and *Lactococcus* were the most representative differentially abundant microorganisms in the high_metabolism group (Fig. 8C). Intergroup difference analysis revealed that, at the genus level, the relative abundances of *Lactococcus* and *Defluviitaleaceae_UCG-011* were significantly higher in the high_metabolism group (Fig. S6C, *P* < 0.05). At the species level, the relative abundances of *Eubacterium_hallii_g_Eubacterium_hallii_group*, *uncultured_marine_bacterium_g_Christensenellaceae_R-7 group* and *uncultured_bacterium_g_Defluviitaleaceae_UCG-011* were significantly increased in the high_metabolism group (Fig. 8D, *P* < 0.05). Correlation analysis further revealed a significant positive association between saikosaponin D biotransformation capacity and the relative abundance of specific microbial taxa, including *Defluviitaleaceae_UCG-011* and *Lactococcus* at the genus level, and *Eubacterium_hallii_g_Eubacterium_hallii_group* at the species level (Fig. S7, *P* < 0.05).

**Fig. 9:**
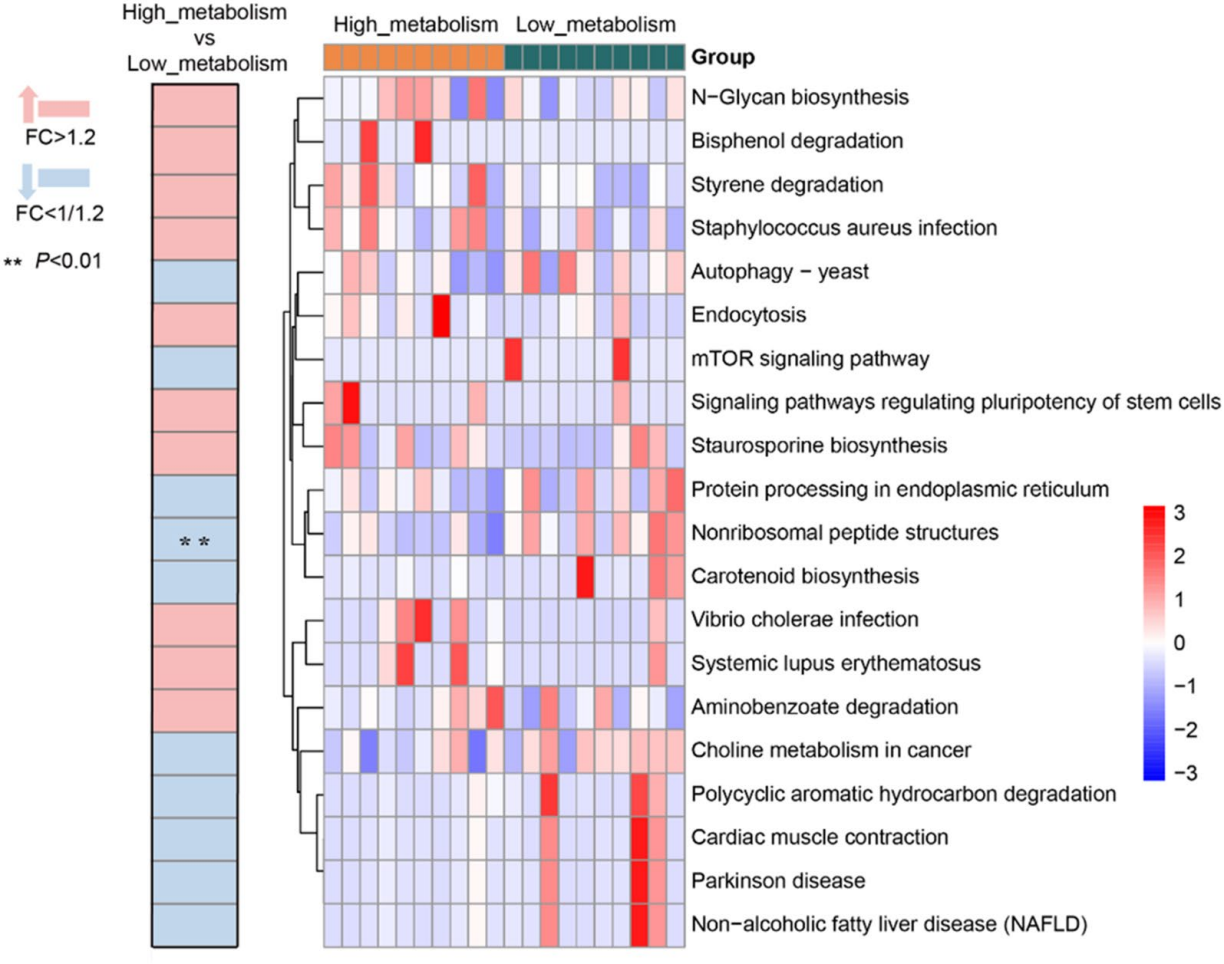
Heatmap summarizing the changes in gut microbial community function based on differences in biotransformation of Saikosaponin D ex vivo. (Pink color represents enrichment in high_metabolism group, blue color represents enrichment in low_metabolism group; ^**^P < 0.01; *n* = 10 per group.)

Differential analysis of KEGG metabolic pathways showed that, except for nonribosomal peptide structures (which exhibited significantly reduced relative abundance in the high-metabolism group; Fig. 9, *P* < 0.05), gut microbiota metabolic pathways did not differ significantly between the two groups. This result aligns with the finding that the biotransformation activity of saikosaponin D by the gut microbiota from 50 healthy volunteers showed relatively low inter-individual variability (Fig. 3B).

**Fig. 10:**
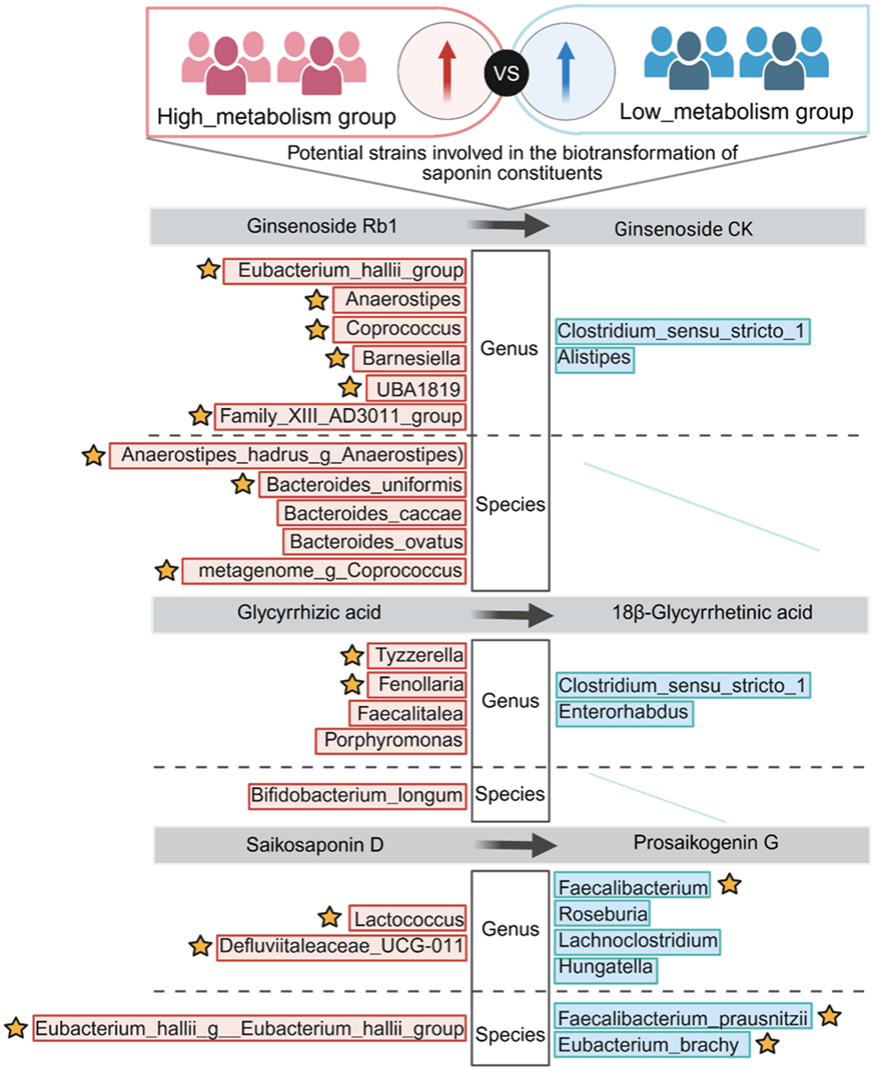
Differential species summary of human gut microbiota based on differences in biotransformation of three saponin components (Ginsenoside Rb1, Glycyrrhizic acid and Saikosaponin D) ex vivo. (Pink color represents strains significantly up-regulated in high_metabolism group and blue color represents strains significantly up-regulated in low_metabolism group; Asterisks indicate microbial taxa that were significantly associated with saponins biotransformation;* P* < 0.05.)

In summary, these results suggested that the higher biotransformation capacity of the three saponins by the gut microbiota of the high_metabolism group may be associated with differences in gut microbiota composition and diversity between the high_ and low_metabolism groups. The specific microbial taxa with higher relative abundance in the high_metabolism group (Fig. 10), particularly those significantly associated with biotransformation of saponins (marked with asterisks), are considered potential key factors to this difference and represent potential targets for further mechanistic investigation. Additionally, the observed microbiota compositional and diversity differences may be closely related to their metabolic functions.

## Discussion

Our current study, building upon the previously established “host in situ gut microbiota culture” technology [25], systematically evaluated the species-specific differences and inter-individual variability in gut microbiota mediated biotransformation of saponins. The results revealed distinct biotransformation patterns of multi-component saponins by mixed gut microbiota, with notable inter-individual variability observed among 50 healthy volunteers. Particularly, ginsenoside Rb1 exhibited the most significant individual differences in gut microbiota biotransformation. Further analysis indicated a potential association between the biotransformation capacity of gut microbiota and its taxonomic composition, possibly mediated by the relative abundances of specific bacterial genera.

Previous studies have demonstrated that gut microbiota mediates the stepwise deglycosylation of saponins through secretion of glycoside hydrolases [26, 27], while suggesting species-specific differences in these biotransformation processes [10, 11]. However, existing research has been predominantly confined to analyses of single bacterial species or specific saponin. In this study, we systematically evaluated the metabolic profiles of mixed gut microbiota (derived from human and mouse sources, respectively) on five representative saponins (ginsenoside Rb1, ginsenoside Re, glycyrrhizic acid, saikosaponin D and dioscin). Our findings not only corroborated the previously reported species-specific differences, but further revealed that three saponins (ginsenoside Rb1, glycyrrhizic acid and saikosaponin D) exhibited no discernible effects on the growth of gut microbiota derived from either humans or mice under ex vivo conditions. This observation suggests that the underlying mechanisms for species-specific differences regarding gut microbiota-mediated saponin biotransformation can be explored by focusing on differences in gut microbiota composition and functional networks. Furthermore, by constructing a cross-species comparative model, we analyzed differences in microbiota-dependent biotransformation, a pivotal factor in translational medicine research. This provides a theoretical basis at the microbiome level for optimizing the research paradigm of translational medicine and helps to enhance the biological relevance of clinical research design.

Inter-individual variability in drug response represents a common clinical phenomenon, with increasing recognition of gut microbiota-mediated personalized drug metabolism profiles in recent years. Previous investigations have revealed substantial inter-individual variability in the biotransformation of herbal constituents by human fecal microbiota [9, 28, 29]. The differences in the human gut microbiome may account for a significant proportion of variable drug responses that had previously been ascribed primarily to polymorphisms in drug-metabolizing genes [30, 31]. Our results indicated that ginsenoside Rb1, glycyrrhizic acid and saikosaponin D were metabolized by the gut microbiota of 50 healthy volunteers, consistent with the aforementioned findings. Moreover, the biotransformation of the three saponins exhibited varying degrees of inter-individual variability, with ginsenoside Rb1 demonstrating the most pronounced metabolic differences. However, the metabolic activities of gut microbiota, such as deglycosylation of saponins, are closely associated with their species composition and the relative abundance of different genera [32–34]. In this study, we found that individuals with high biotransformation capacity for the three saponins exhibited higher species diversity and richness in their gut microbiota compared to those with low biotransformation capacity, particularly for ginsenoside Rb1. Previous studies have shown that the microbiota involved in the biotransformation of saponins primarily belong to genera such as *Bifidobacterium*, *Eubacterium*, *Enterococcus* and *Escherichia* [27], which are rich in enzymes capable of hydrolyzing glycosidic bonds. Our findings further demonstrated that the relative abundance of *Eubacterium_hallii_group* was significantly higher in the high_metabolism group than in the low_metabolism group for the biotransformation of ginsenoside Rb1. In addition, the relative abundance of *Bifidobacterium*_*longum* from the genus *Bifidobacterium* was significantly higher in the high_metabolism group in the biotransformation of glycyrrhizic acid. In the analysis of human gut microbiota for differences in biotransformation of saikosaponin D, the relative abundance of *Eubacterium_hallii_g_Eubacterium hallii_group* from the genus *Eubacterium_hallii_group* was significantly higher in the high_metabolism group. Furthermore, significant inter-individual variability exists in gut microbiota functions and metabolic pathways, which aligns with the characteristic abundance of metabolism-related genes within microbial communities. Notably, host genetics, dietary patterns, and environmental factors collectively regulate both the composition of gut microbiota and the distribution of functional genes [35, 36], resulting in substantial individual differences in metabolic enzyme profiles and catalytic activities. These variations may directly influence the efficiency of saponin biotransformation and the spectrum of resultant metabolites.

This study has several limitations. First, while we quantified key bioactive metabolites to assess inter-species and inter-individual differences in saponin biotransformation, comprehensive metabolic profiling was not performed, which could reveal additional transformation pathways. Second, although we identified microbiota-dependent metabolic patterns, their pharmacological implications were inferred from literature rather than validated experimentally; future studies should integrate in vitro and in vivo models to directly link biotransformation phenotypes to functional outcomes. Third, despite analyzing 50 samples, the high heterogeneity of gut microbiota and structural diversity of saponins may require larger cohorts to identify conserved microbial taxa or enzymes driving specific reactions. Finally, while correlations between microbial features and biotransformation capacity were observed, causal mechanisms require validation through functional genomics or enzyme activity assays. These gaps highlight the need for multi-omics approaches coupled with mechanistic studies to fully elucidate microbiota-saponin interactions.

## Conclusion

Our study advances the understanding of gut microbiota-saponins interactions through dual perspectives of species-specific differences and inter-individual variability. We elucidated the pivotal role of gut microbiota in mediating inter-individual differences in saponin biotransformation, and identified potential microbial communities and metabolic pathways involved in this process. These findings provide a scientific foundation for the subsequent screening of suitable strains or enzymes that can metabolize saponins from human gut microbiota to optimize saponin-based therapeutic regimens. Given the shared enzymatic mechanisms across natural products, this approach may extend to other natural products, underscoring the potential of microbiome-personalized therapies in precision medicine.

## Supplementary Information

Supplementary Material 1.

## Data Availability

Data is provided within the manuscript file. Find some help on our Data availability statements page.

## Abbreviations

LefSe: Linear discriminant analysis effect size
PCoA: Principal coordinates analysis
UPLC-MS/MS: Ultra performance liquid chromatography tandem mass spectrometry

## References

[1] Zimmermann M, Zimmermann-Kogadeeva M, Wegmann R, Goodman AL. Mapping human microbiome drug metabolism by gut bacteria and their genes. Nature. 2019;570(7762):462–7. 31158845 10.1038/s41586-019-1291-3 PMC6597290

[2] Liao CP, Liu XC, Dong SQ, An M, Zhao L, Zhang AJ, et al. Investigation of the metabolites of five major constituents from *Berberis amurensis* in normal and pseudo germ-free rats. Chin J Nat Med. 2021;19(10):758–71. 34688466 10.1016/S1875-5364(21)60082-1

[3] Guo YP, Chen MY, Shao L, Zhang W, Rao T, Zhou HH, et al. Quantification of *Panax notoginseng* saponins metabolites in rat plasma with in vivo gut microbiota-mediated biotransformation by HPLC-MS/MS. Chin J Nat Med. 2019;17(3):231–40. 30910060 10.1016/S1875-5364(19)30026-3

[4] Walsh J, Griffin BT, Clarke G, Hyland NP. Drug-gut microbiota interactions: implications for neuropharmacology. Br J Pharmacol. 2018;175(24):4415–29. 29782640 10.1111/bph.14366 PMC6255959

[5] Zhang J, Zhang J, Wang R. Gut microbiota modulates drug pharmacokinetics. Drug Metab Rev. 2018;50(3):357–68. 30227749 10.1080/03602532.2018.1497647

[6] Kim DH. Gut microbiota-mediated pharmacokinetics of ginseng saponins. J Ginseng Res. 2018;42(3):255–63. 29983606 10.1016/j.jgr.2017.04.011 PMC6026358

[7] Bae EA, Park SY, Kim DH. Constitutive beta-glucosidases hydrolyzing ginsenoside Rb1 and Rb2 from human intestinal bacteria. Biol Pharm Bull. 2000;23(12):1481–5. 11145182 10.1248/bpb.23.1481

[8] Yan X, Bai X, Fu R, Duan Z, Zeng W, Zhu C. Ginsenoside compound K alleviates D-galactose-induced mild cognitive impairment by modulating gut microbiota-mediated short-chain fatty acid metabolism. Food Funct. 2024;15(18):9037–52. 39150321 10.1039/d4fo03216k

[9] Kim YS, Kim JJ, Cho KH, Jung WS, Moon SK, Park EK, et al. Biotransformation of ginsenoside Rb1, crocin, amygdalin, geniposide, puerarin, ginsenoside Re, hesperidin, poncirin, glycyrrhizin, and baicalin by human fecal microflora and its relation to cytotoxicity against tumor cells. J Microbiol Biotechnol. 2008;18(6):1109–14. 18600055

[10] Cao WY, Wang YN, Wang PY, Lei W, Feng B, Wang XJ. Ardipusilloside-I metabolites from human intestinal bacteria and their antitumor activity. Molecules. 2015;20(11):20569–81. 26610438 10.3390/molecules201119719 PMC6331786

[11] Wang XJ, Cui H, Wang R, Huan ML, Zhang BL, Zhang WD, et al. Metabolism and pharmacokinetic study of ardipusilloside I in rats. Planta Med. 2012;78(6):565–74. 22307936 10.1055/s-0031-1298238

[12] Javdan B, Lopez JG, Chankhamjon P, Lee YJ, Hull R, Wu Q, et al. Personalized mapping of drug metabolism by the human gut microbiome. Cell. 2020;181(7):1661-79 e22. 32526207 10.1016/j.cell.2020.05.001 PMC8591631

[13] Chen L, Wang D, Garmaeva S, Kurilshikov A, Vich Vila A, Gacesa R, et al. The long-term genetic stability and individual specificity of the human gut microbiome. Cell. 2021;184(9):2302-15.e12. 33838112 10.1016/j.cell.2021.03.024

[14] Li M, Li H. Microbial-host-isozyme: a new territory for understanding personalized responses towards drug therapy. Chin J Nat Med. 2023;21(8):561–2. 37611974 10.1016/S1875-5364(23)60493-5

[15] Li J, Li F, Jin D. Ginsenosides are promising medicine for tumor and inflammation: a review. Am J Chin Med. 2023;51(4):883–908. 37060192 10.1142/S0192415X23500416

[16] Cui X, Ma X, Li C, Meng H, Han C. A review: structure-activity relationship between saponins and cellular immunity. Mol Biol Rep. 2023;50(3):2779–93. 36583783 10.1007/s11033-022-08233-z

[17] Luo Z, Yin F, Wang X, Kong L. Progress in approved drugs from natural product resources. Chin J Nat Med. 2024;22(3):195–211. 38553188 10.1016/S1875-5364(24)60582-0

[18] Jeon JH, Lee J, Choi MK, Song IS. Pharmacokinetics of ginsenosides following repeated oral administration of red ginseng extract significantly differ between species of experimental animals. Arch Pharm Res. 2020;43(12):1335–46. 33225388 10.1007/s12272-020-01289-0

[19] Hugenholtz F, de Vos WM. Mouse models for human intestinal microbiota research: a critical evaluation. Cell Mol Life Sci. 2018;75(1):149–60. 29124307 10.1007/s00018-017-2693-8 PMC5752736

[20] Guo YP, Shao L, Wang L, Chen MY, Zhang W, Huang WH. Bioconversion variation of ginsenoside CK mediated by human gut microbiota from healthy volunteers and colorectal cancer patients. Chin Med. 2021;16(1):28. 33731196 10.1186/s13020-021-00436-z PMC7968294

[21] Wang L, Chen MY, Shao L, Zhang W, Li XP, Huang WH. Personalized bioconversion of *Panax notoginseng* saponins mediated by gut microbiota between two different diet-pattern healthy subjects. Chin Med. 2021;16(1):60. 34301288 10.1186/s13020-021-00476-5 PMC8306348

[22] Huang S, Shao L, Chen M, Wang L, Liu J, Zhang W, et al. Biotransformation differences of ginsenoside compound K mediated by the gut microbiota from diabetic patients and healthy subjects. Chin J Nat Med. 2023;21(10):723–9. 37879791 10.1016/S1875-5364(23)60402-9

[23] Yuan M, Shi DZ, Wang TY, Zheng SQ, Liu LJ, Sun ZX, et al. Transformation of trollioside and isoquercetin by human intestinal flora *in vitro*. Chin J Nat Med. 2016;14(3):220–6. 27025369 10.1016/S1875-5364(16)30019-X

[24] Shen H, Leung WI, Ruan JQ, Li SL, Lei JP, Wang YT, et al. Biotransformation of ginsenoside Rb1 via the gypenoside pathway by human gut bacteria. Chin Med. 2013;8(1):22. 24267405 10.1186/1749-8546-8-22 PMC4175505

[25] Tao X, Huang W, Pan L, Sheng L, Qin Y, Chen L, et al. Optimizing ex vivo culture conditions to study human gut microbiome. ISME Commun. 2023;3(1): 38. 37185811 10.1038/s43705-023-00245-5 PMC10130157

[26] Jia B, Han X, Kim KH, Jeon CO. Discovery and mining of enzymes from the human gut microbiome. Trends Biotechnol. 2022;40(2):240–54. 34304905 10.1016/j.tibtech.2021.06.008

[27] Xu J, Chen HB, Li SL. Understanding the molecular mechanisms of the interplay between herbal medicines and gut microbiota. Med Res Rev. 2017;37(5):1140–85. 28052344 10.1002/med.21431

[28] Lee DS, Kim YS, Ko CN, Cho KH, Bae HS, Lee KS, et al. Fecal metabolic activities of herbal components to bioactive compounds. Arch Pharm Res. 2002;25(2):165–9. 12009030 10.1007/BF02976558

[29] Yim JS, Kim YS, Moon SK, Cho KH, Bae HS, Kim JJ, et al. Metabolic activities of ginsenoside Rb1, baicalin, glycyrrhizin and geniposide to their bioactive compounds by human intestinal microflora. Biol Pharm Bull. 2004;27(10):1580–3. 15467199 10.1248/bpb.27.1580

[30] Schupack DA, Mars RAT, Voelker DH, Abeykoon JP, Kashyap PC. The promise of the gut microbiome as part of individualized treatment strategies. Nat Rev Gastroenterol Hepatol. 2022;19(1):7–25. 34453142 10.1038/s41575-021-00499-1 PMC8712374

[31] Li H, He J, Jia W. The influence of gut microbiota on drug metabolism and toxicity. Expert Opin Drug Metab Toxicol. 2016;12(1):31–40. 26569070 10.1517/17425255.2016.1121234 PMC5683181

[32] Kim JH, Oh JM, Chun S, Park HY, Im WT. Enzymatic biotransformation of ginsenoside Rb(2) into Rd by recombinant α-L-arabinopyranosidase from Blastococcus saxobsidens. J Microbiol Biotechnol. 2020;30(3):391–7. 31893597 10.4014/jmb.1910.10065 PMC9728169

[33] Chang KH, Jo MN, Kim KT, Paik HD. Purification and characterization of a ginsenoside Rb(1)-hydrolyzing β-glucosidase from *Aspergillus niger* KCCM 11239. Int J Mol Sci. 2012;13(9):12140–52. 23109906 10.3390/ijms130912140 PMC3472798

[34] Hu Y, Zhai L, Hong H, Shi Z, Zhao J, Liu D. Study on the biochemical characterization and selectivity of three β-glucosidases from *Bifidobacterium adolescentis* ATCC15703. Front Microbiol. 2022;13: 860014. 35464910 10.3389/fmicb.2022.860014 PMC9024363

[35] David LA, Maurice CF, Carmody RN, Gootenberg DB, Button JE, Wolfe BE, et al. Diet rapidly and reproducibly alters the human gut microbiome. Nature. 2014;505(7484):559–63. 24336217 10.1038/nature12820 PMC3957428

[36] Goodrich JK, Waters JL, Poole AC, Sutter JL, Koren O, Blekhman R, et al. Human genetics shape the gut microbiome. Cell. 2014;159(4):789–99. 25417156 10.1016/j.cell.2014.09.053 PMC4255478

