# Supplementary material for: Inter-species and individualized biotransformation of five saponins by human being- and mouse-derived fecal microbiota
Source: Chin Med. 2025 Aug 25;20:132. doi: 10.1186/s13020-025-01190-2 (PMC12376353; doi:10.1186/s13020-025-01190-2)
Supplement: Supplementary file 1 — Supplementary Material 1. [file 13020_2025_1190_MOESM1_ESM.docx]

**Table S1. Baseline information for 50 donors**

| Donor | Age | Gender | Ginsenoside Rb1 | Glycyrrhizic acid | Saikosaponin D |
| --- | --- | --- | --- | --- | --- |
| 1 | 28 | male | / | / | / |
| 2 | 24 | female | / | High_metabolism | / |
| 3 | 24 | male | / | High_metabolism | / |
| 4 | 30 | female | / | High_metabolism | / |
| 5 | 24 | male | / | High_metabolism | / |
| 6 | 24 | female | / | High_metabolism | High_metabolism |
| 7 | 19 | female | High_metabolism | High_metabolism | High_metabolism |
| 8 | 23 | female | High_metabolism | / | / |
| 9 | 21 | female | / | High_metabolism | / |
| 10 | 26 | female | Low_metabolism | Low_metabolism | / |
| 11 | 25 | female | / | / | / |
| 12 | 21 | male | High_metabolism | High_metabolism | / |
| 13 | 25 | female | Low_metabolism | Low_metabolism | High_metabolism |
| 14 | 24 | female | / | High_metabolism | Low_metabolism |
| 15 | 19 | female | / | / | / |
| 16 | 22 | male | / | / | / |
| 17 | 24 | female | High_metabolism | / | High_metabolism |
| 18 | 18 | male | / | / | High_metabolism |
| 19 | 30 | male | High_metabolism | / | High_metabolism |
| 20 | 24 | male | Low_metabolism | Low_metabolism | / |
| 21 | 25 | female | / | / | Low_metabolism |
| 22 | 22 | female | / | / | / |
| 23 | 23 | male | Low_metabolism | / | Low_metabolism |
| 24 | 29 | male | Low_metabolism | Low_metabolism | / |
| 25 | 24 | male | / | / | / |
| 26 | 27 | female | / | / | Low_metabolism |
| 27 | 23 | female | High_metabolism | / | / |
| 28 | 23 | female | / | / | / |
| 29 | 25 | male | High_metabolism | Low_metabolism | High_metabolism |
| 30 | 26 | female | Low_metabolism | Low_metabolism | / |
| 31 | 23 | male | / | / | / |
| 32 | 20 | male | / | / | / |
| 33 | 21 | male | / | / | Low_metabolism |
| 34 | 22 | male | / | / | Low_metabolism |
| 35 | 21 | male | / | Low_metabolism | High_metabolism |
| 36 | 24 | female | / | High_metabolism | / |
| 37 | 23 | male | / | / | / |
| 38 | 22 | male | High_metabolism | / | Low_metabolism |
| 39 | 21 | male | Low_metabolism | / | Low_metabolism |
| 40 | 22 | male | High_metabolism | / | High_metabolism |
| 41 | 24 | male | High_metabolism | Low_metabolism | High_metabolism |
| 42 | 24 | female | Low_metabolism | / | / |
| 43 | 23 | female | Low_metabolism | Low_metabolism | / |
| 44 | 27 | female | / | / | Low_metabolism |
| 45 | 25 | male | / | / | / |
| 46 | 25 | female | / | / | Low_metabolism |
| 47 | 25 | female | Low_metabolism | / | / |
| 48 | 26 | male | / | Low_metabolism | / |
| 49 | 31 | male | / | / | / |
| 50 | 20 | female | / | / | / |

**Table S2. The chemicals, media, and reagents**

| Reagent or resource | Source | Identifier |
| --- | --- | --- |
| Chemicals, media and reagents | | |
| Casein enzymic hydrolysate | Sangon | A10085-0100 |
| Dextrose | Sangon | A610291 |
| Digested serum | YuanMu | YR0305 |
| Hemin | Sigma-Aldrich | H9039-1G |
| KH_2_PO_4_ | Sinopharm | 10017618 |
| L-Arginine | Sangon | A600205-0100 |
| L-Cysteine hydrochloride | Sinopharm | XW00528911 |
| Liver extract | Hongrun Baoshun | Y037 |
| L-Tryptophan | Sinopharm | 73-22-3 |
| Beef extract | Sangon | A600114-0100 |
| NaCl | Sinopharm | 10019318 |
| Peptone | Sangon | A100636-0100 |
| Proteose peptone | Sangon | A600241-0100 |
| Resazurin | Sangon | A606726-0001 |
| Sodium acetate | Sigma-Aldrich | S8750-250G |
| Soya peptone | Sangon | A600214-0100 |
| Soluble starch | Sangon | A500904-0500 |
| Sodium thioglycolate | Sangon | A610265-0025 |
| Vitamin K1 | Sangon | A606528-0005 |
| Yeast extract | BD-AB | 212750 |
| PBS(10×) | Beyotime | ST476 |
| L-Cysteine | Vetec | V900400-100G |
| Drugs | | |
| Ginsenoside Rb1 | DeSiTe | DSTDR000603 |
| Ginsenoside Re | DeSiTe | DSTDR001401 |
| Glycyrrhizic acid | DeSiTe | DSTDG000604 |
| Saikosaponin D | DeSiTe | DSTDC000802 |
| Dioscin | DeSiTe | DSTDS000501 |
| Ginsenoside CK | DeSiTe | DSTDR003001 |
| (20S)-Protopanaxatriol | DeSiTe | DST210827-461 |
| 18β-Glycyrrhetinic acid | DeSiTe | DSTDG000702 |
| Prosaikogenin G | DeSiTe | DST230824-232 |
| Diosgenin | DeSiTe | DSTDS001202 |

**Table S3. The composition of GB culture media**

| Composition Name | Concentration ( g/L ) | | | |
| --- | --- | --- | --- | --- |
|  | GB | | | |
|  | mGAM | | BB | |
| Dextrose | 0.50 | PH=7.3±0.1 | / | PH=5.9±0.2 |
| Soluble Starch | 5.00 |  |  |  |
| Yeast Extract | 2.50 |  | 5.00 |  |
| Meat Extract | 2.20 |  | 7.50 |  |
| Liver Extract | 1.20 |  | / |  |
| Digested Serum | 10.00 |  |  |  |
| Peptone | 5.00 |  |  |  |
| Soya peptone | 3.00 |  |  |  |
| Proteose Peptone | 5.00 |  |  |  |
| L-Tryptophan | 0.20 |  |  |  |
| L-Arginine | 1.00 |  |  |  |
| L-Cysteine Hydrochloride | 0.30 |  | 0.50 |  |
| Casein enzymic hydrolysate | / |  | 15.00 |  |
| Potassium Dihydrogen Phosphate | 2.50 |  | / |  |
| Sodium Thioglycolate | 0.30 |  |  |  |
| Sodium chloride | 3.00 |  |  |  |
| Sodium acetate | / |  | 5.00 |  |
| Vitamin K1 | 0.0050 |  | / |  |
| Hemin | 0.0100 |  |  |  |
| Resazurin | / |  | 0.0025 |  |

| Compound | Q1 (Da) | Q3 (Da) | DP (V) | CE (eV) | EP (V) | CXP (V) |
| --- | --- | --- | --- | --- | --- | --- |
| Ginsenoside Rb1 | 1153.6 | 1107.5 | -180 | -31 | -10 | -19 |
| Ginsenoside Re | 991.4 | 945.3 | -35 | -35 | -10 | -11 |
| Glycyrrhizic acid | 821.4 | 351.2 | -130 | -56 | -10 | -17 |
| Saikosaponin D | 825.5 | 779.2 | -152 | -30 | -10 | -21 |
| Dioscin | 913.4 | 867.6 | -60 | -30 | -10 | -13 |
| Ginsenoside CK | 667.6 | 621.4 | -80 | -28 | -10 | -11 |
| (20S)-Protopanaxatriol | 475.3 | 391.4 | -130 | -42 | -10 | -13 |
| 18β-Glycyrrhetinic acid | 469.3 | 354.9 | -80 | -69 | -10 | -32 |
| Prosaikogenin G | 663.3 | 617.2 | -30 | -33 | -10 | -17 |
| Diosgenin | 415.4 | 271.1 | 60 | 26 | 10 | 10 |

**Table S4. MRM transitions and the optimum UPLC–MS/MS conditions**


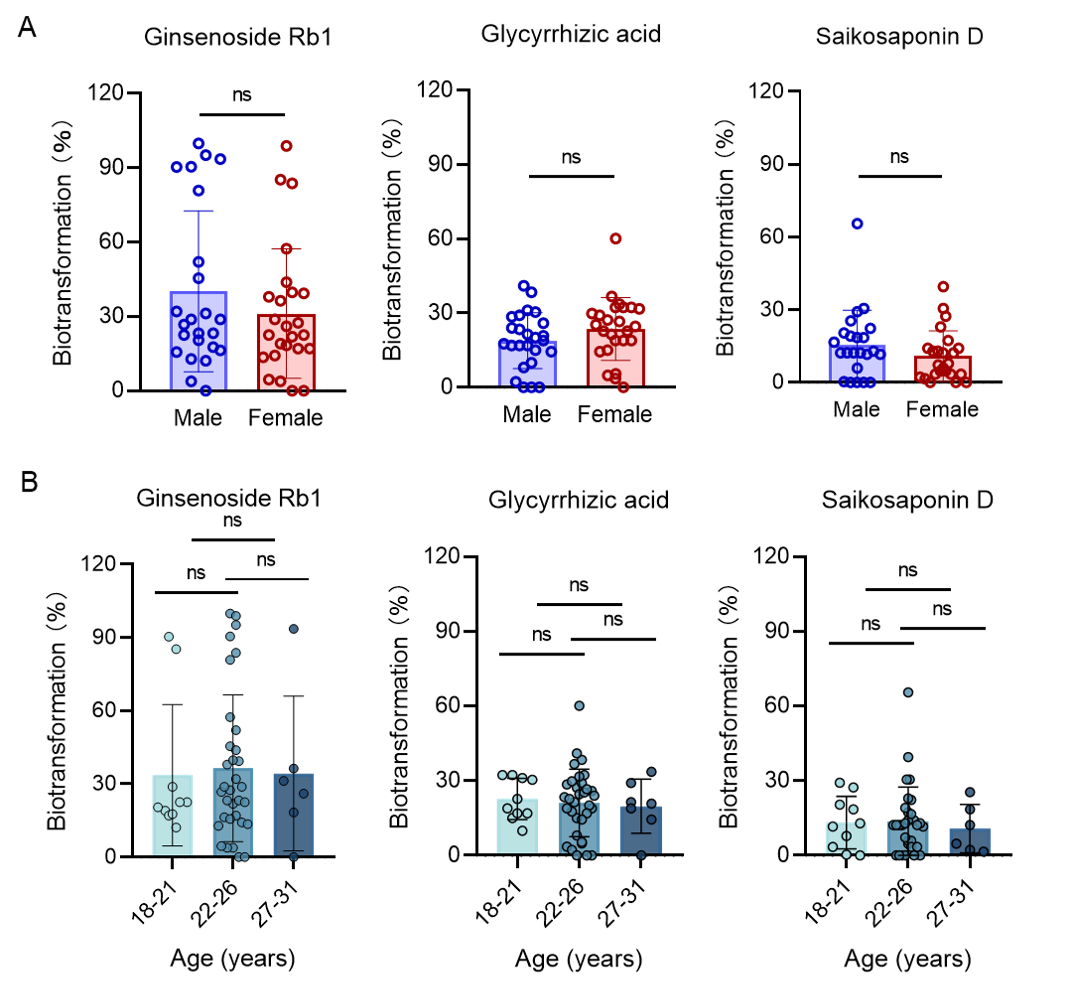


**Fig. S1 Exploratory analysis of donor sex and age associations with biotransformation of saponins.** (A) Scatter plot with bar comparing sex-specific differences in biotransformation. (B) Scatter plot with bar comparing age-specific differences in biotransformation. Prototype drug biotransformation (%) was quantified as (C_0 h_ - C_24 h_)/C_0 h_ × 100%, where C_0 h_ and C_24 h_ represent the prototype drug concentrations at 0 h and 24 h, respectively. Data are presented as mean ± SEM. Statistical significance was assessed by one-way ANOVA, followed by Tukey’s post hoc test.


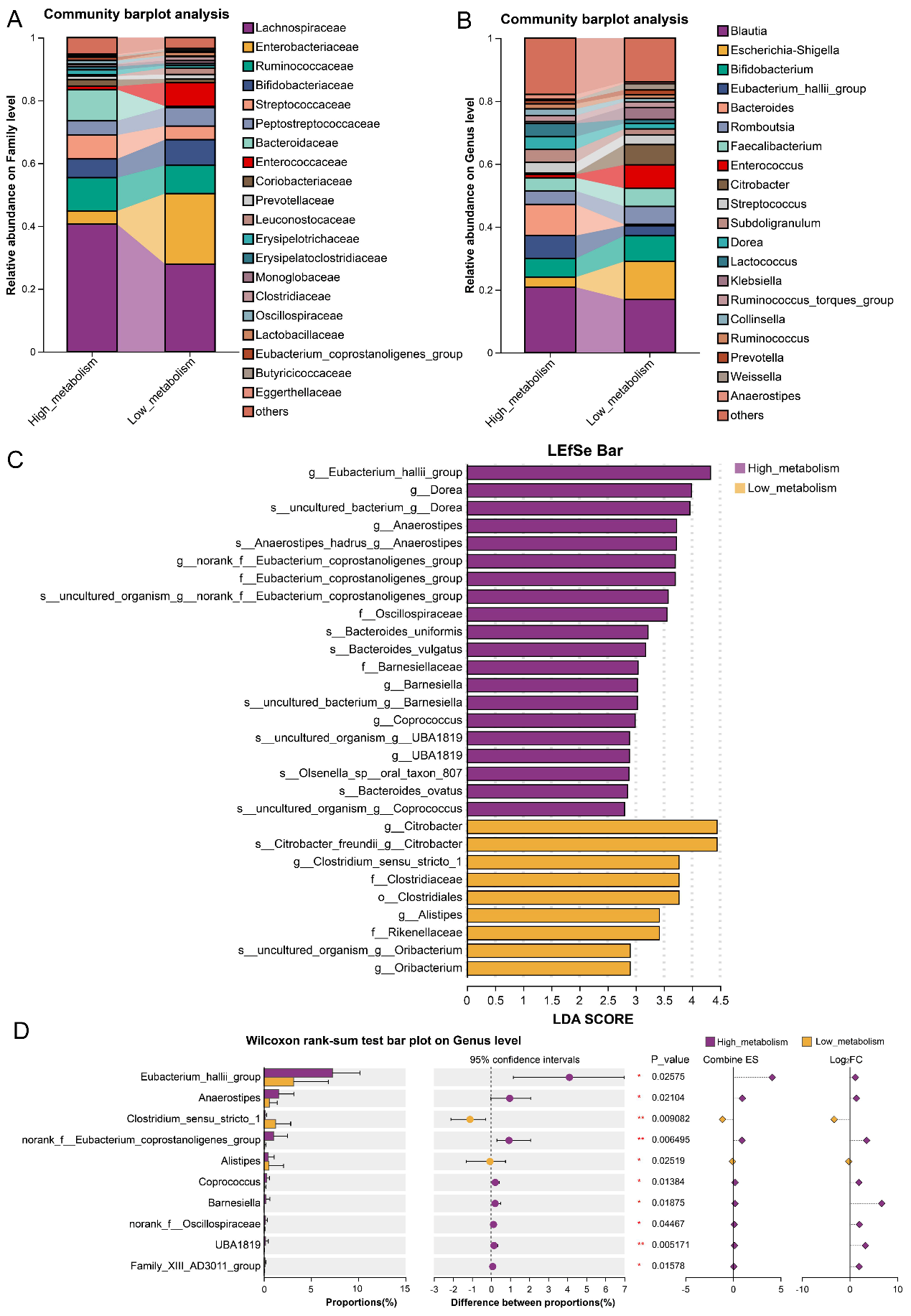
 **Fig. S2 Analysis of human gut microbiota based on differences in biotransformation of Ginsenoside Rb1 *ex vivo*.** (A) Gut microbiota change at Family level (barplot). (B) Gut microbiota change at Genus level (barplot). (C) Gut microbiota structure difference LEfSe analysis. (D) Differentially abundant genus (Wilcoxon rank-sum test). Exact P-values and 95% CIs are indicated in the figure. *^*^P*<0.05; *n*=10 per group.


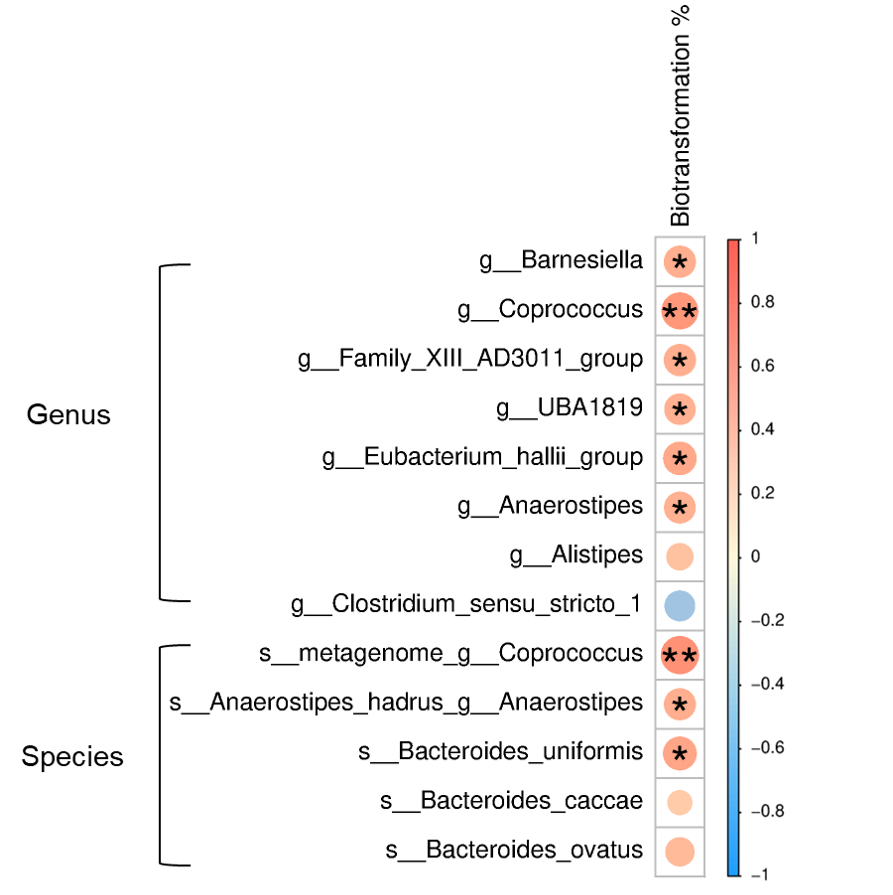


**Fig. S3 Spearman’s correlation analysis between the biotransformation of Ginsenoside Rb1 and specific microbial taxa.** Larger red points indicate greater positive correlation; larger blue points indicate greater negative correlation. An asterisk (*) on the dot indicates the P-value, *^*^P* < 0.05, *^**^P* < 0.01.


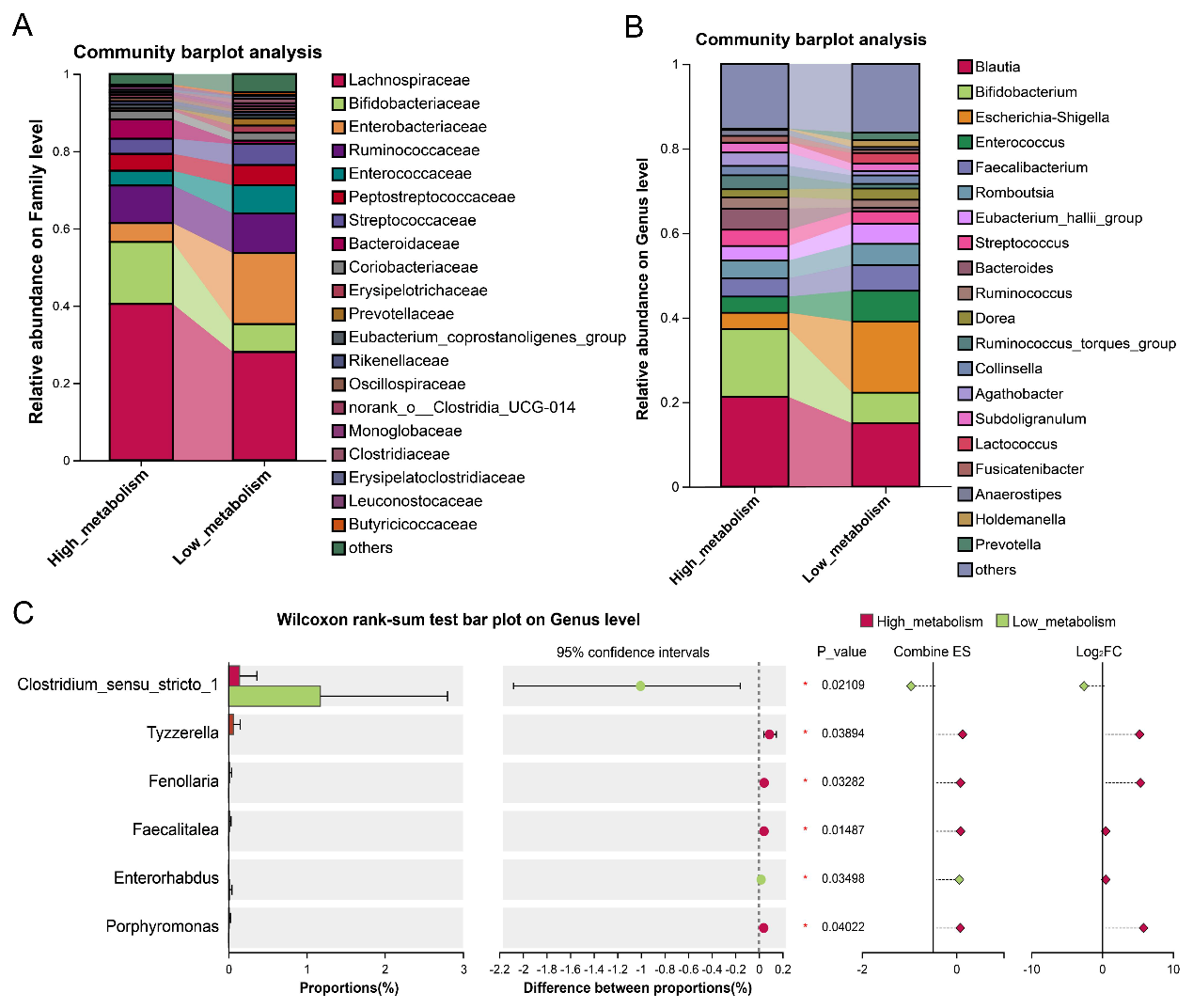
 **Fig. S4 Analysis of human gut microbiota based on differences in biotransformation of Glycyrrhizic acid *ex vivo*.** (A) Gut microbiota change at Family level (barplot). (B) Gut microbiota change at Genus level (barplot). (C) Differentially abundant genus (Wilcoxon rank-sum test). Exact P-values and 95% CIs are indicated in the figure. *^*^P*<0.05; *n*=10 per group.

**
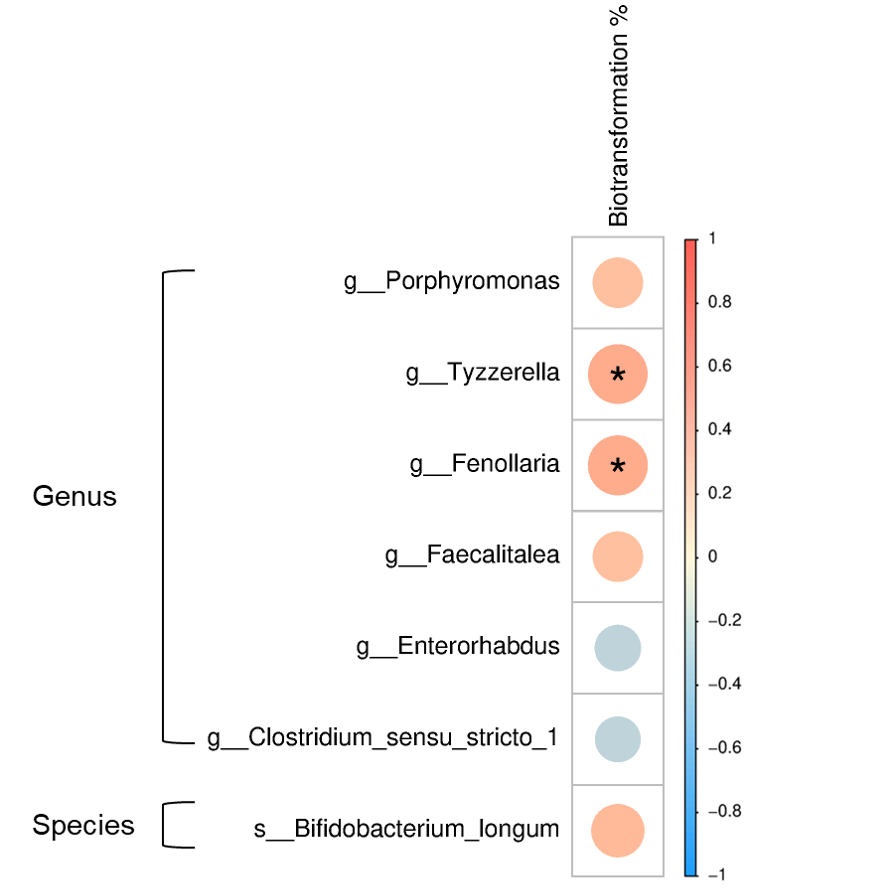
**

**Fig.** **S5 Spearman’s correlation analysis between the biotransformation of Glycyrrhizic acid and specific microbial taxa.** Larger red points indicate greater positive correlation; larger blue points indicate greater negative correlation. An asterisk (*) on the dot indicates the P-value, *^*^P* < 0.05.


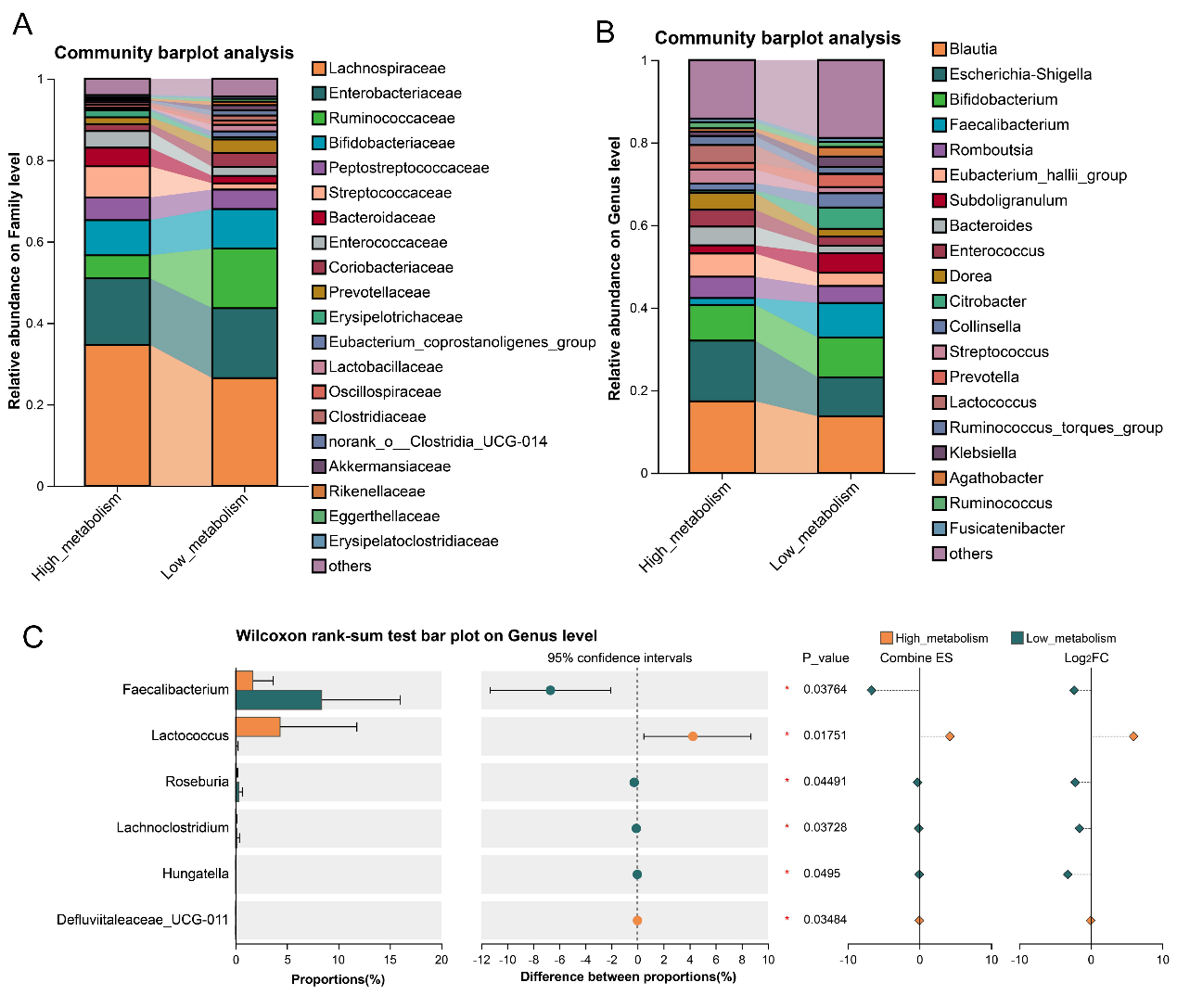
 **Fig. S6 Analysis of human gut microbiota based on differences in biotransformation of Saikosaponin D *ex vivo*.** (A) Gut microbiota change at Family level (barplot). (B) Gut microbiota change at Genus level (barplot). (C) Differentially abundant genus (Wilcoxon rank-sum test). Exact P-values and 95% CIs are indicated in the figure. *^*^P*<0.05; *n*=10 per group.


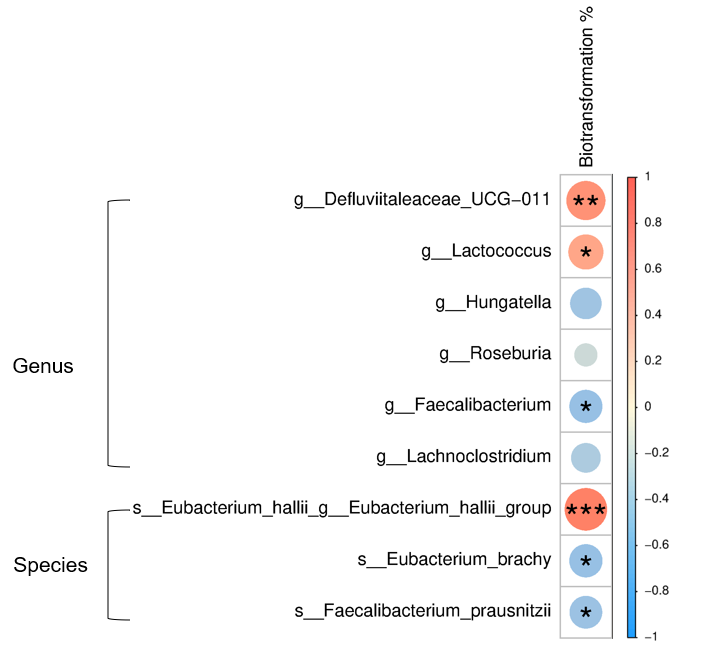


**Fig. S7 Spearman’s correlation analysis between the biotransformation of Saikosaponin D and specific microbial taxa.** Larger red points indicate greater positive correlation; larger blue points indicate greater negative correlation. An asterisk (*) on the dot indicates the P-value, *^*^P* < 0.05, *^**^P* < 0.01, *^***^P* < 0.001.
